# Oxidative phosphorylation safeguards pluripotency via UDP-*N*-acetylglucosamine

**DOI:** 10.1093/procel/pwac009

**Published:** 2022-10-14

**Authors:** Jiani Cao, Meng Li, Kun Liu, Xingxing Shi, Ning Sui, Yuchen Yao, Xiaojing Wang, Shiyu Li, Yuchang Tian, Shaojing Tan, Qian Zhao, Liang Wang, Xiahua Chai, Lin Zhang, Chong Liu, Xing Li, Zhijie Chang, Dong Li, Tongbiao Zhao

**Affiliations:** State Key Laboratory of Stem Cell and Reproductive Biology, Institute for Stem Cell and Regeneration, Institute of Zoology, Chinese Academy of Sciences, Beijing 100101, China; Beijing Institute for Stem Cell and Regenerative Medicine, Beijing 100101, China; State Key Laboratory of Membrane Biology, School of Medicine, Center for Synthetic and Systems Biology, Tsinghua University, Beijing 100084, China; State Key Laboratory of Stem Cell and Reproductive Biology, Institute for Stem Cell and Regeneration, Institute of Zoology, Chinese Academy of Sciences, Beijing 100101, China; Beijing Institute for Stem Cell and Regenerative Medicine, Beijing 100101, China; State Key Laboratory of Stem Cell and Reproductive Biology, Institute for Stem Cell and Regeneration, Institute of Zoology, Chinese Academy of Sciences, Beijing 100101, China; Beijing Institute for Stem Cell and Regenerative Medicine, Beijing 100101, China; University of Chinese Academy of Sciences, Beijing 100049, China; State Key Laboratory of Stem Cell and Reproductive Biology, Institute for Stem Cell and Regeneration, Institute of Zoology, Chinese Academy of Sciences, Beijing 100101, China; Beijing Institute for Stem Cell and Regenerative Medicine, Beijing 100101, China; Qufu Normal University, Qufu 273165, China; State Key Laboratory of Stem Cell and Reproductive Biology, Institute for Stem Cell and Regeneration, Institute of Zoology, Chinese Academy of Sciences, Beijing 100101, China; Beijing Institute for Stem Cell and Regenerative Medicine, Beijing 100101, China; Qufu Normal University, Qufu 273165, China; State Key Laboratory of Stem Cell and Reproductive Biology, Institute for Stem Cell and Regeneration, Institute of Zoology, Chinese Academy of Sciences, Beijing 100101, China; Beijing Institute for Stem Cell and Regenerative Medicine, Beijing 100101, China; University of Chinese Academy of Sciences, Beijing 100049, China; State Key Laboratory of Stem Cell and Reproductive Biology, Institute for Stem Cell and Regeneration, Institute of Zoology, Chinese Academy of Sciences, Beijing 100101, China; Beijing Institute for Stem Cell and Regenerative Medicine, Beijing 100101, China; University of Chinese Academy of Sciences, Beijing 100049, China; State Key Laboratory of Stem Cell and Reproductive Biology, Institute for Stem Cell and Regeneration, Institute of Zoology, Chinese Academy of Sciences, Beijing 100101, China; Beijing Institute for Stem Cell and Regenerative Medicine, Beijing 100101, China; University of Chinese Academy of Sciences, Beijing 100049, China; State Key Laboratory of Stem Cell and Reproductive Biology, Institute for Stem Cell and Regeneration, Institute of Zoology, Chinese Academy of Sciences, Beijing 100101, China; Beijing Institute for Stem Cell and Regenerative Medicine, Beijing 100101, China; University of Chinese Academy of Sciences, Beijing 100049, China; State Key Laboratory of Stem Cell and Reproductive Biology, Institute for Stem Cell and Regeneration, Institute of Zoology, Chinese Academy of Sciences, Beijing 100101, China; Beijing Institute for Stem Cell and Regenerative Medicine, Beijing 100101, China; State Key Laboratory of Stem Cell and Reproductive Biology, Institute for Stem Cell and Regeneration, Institute of Zoology, Chinese Academy of Sciences, Beijing 100101, China; Beijing Institute for Stem Cell and Regenerative Medicine, Beijing 100101, China; University of Chinese Academy of Sciences, Beijing 100049, China; State Key Laboratory of Stem Cell and Reproductive Biology, Institute for Stem Cell and Regeneration, Institute of Zoology, Chinese Academy of Sciences, Beijing 100101, China; Beijing Institute for Stem Cell and Regenerative Medicine, Beijing 100101, China; State Key Laboratory of Stem Cell and Reproductive Biology, Institute for Stem Cell and Regeneration, Institute of Zoology, Chinese Academy of Sciences, Beijing 100101, China; Beijing Institute for Stem Cell and Regenerative Medicine, Beijing 100101, China; University of Chinese Academy of Sciences, Beijing 100049, China; National Laboratory of Biomacromolecules, CAS Center for Excellence in Biomacromolecules, Institute of Biophysics, Chinese Academy of Sciences, Beijing 100101, China; University of Chinese Academy of Sciences, Beijing 100049, China; State Key Laboratory of Stem Cell and Reproductive Biology, Institute for Stem Cell and Regeneration, Institute of Zoology, Chinese Academy of Sciences, Beijing 100101, China; Beijing Institute for Stem Cell and Regenerative Medicine, Beijing 100101, China; University of Chinese Academy of Sciences, Beijing 100049, China; State Key Laboratory of Membrane Biology, School of Medicine, Center for Synthetic and Systems Biology, Tsinghua University, Beijing 100084, China; National Laboratory of Biomacromolecules, CAS Center for Excellence in Biomacromolecules, Institute of Biophysics, Chinese Academy of Sciences, Beijing 100101, China; University of Chinese Academy of Sciences, Beijing 100049, China; State Key Laboratory of Stem Cell and Reproductive Biology, Institute for Stem Cell and Regeneration, Institute of Zoology, Chinese Academy of Sciences, Beijing 100101, China; Beijing Institute for Stem Cell and Regenerative Medicine, Beijing 100101, China; University of Chinese Academy of Sciences, Beijing 100049, China

**Dear Editor,**

Embryonic stem cells (ESCs) have been assumed to possess immature mitochondria and to favor anaerobic glycolysis over oxidative phosphorylation (OXPHOS) for energy production. This proposition is largely based on the findings that ESCs possess globular mitochondria with blurred cristae, and the facts that ESCs have higher glycolysis activity and lower mitochondrial respiration capacity than somatic cells (Kondoh et al., 2007; Folmes et al., 2011,  2012; Zhang et al., 2012; Xu et al., 2013; Ito and Suda, 2014; Gu et al., 2016). However, recent studies have shown that mitochondrial autophagy and mitochondrial dynamics are pivotal for ESC self-renewal and pluripotency (Todd et al., 2010; Liu et al., 2016, 2020; Wang et al., 2019; Zhong et al., 2019). These studies have raised a fundamental question: what is the contribution and functional mechanism of mitochondrial respiration in pluripotency regulation?

We firstly determined the total cellular and mitochondrial volumes of individual mouse naïve ESCs (ESCs), primed ESCs (EpiLCs), neural stem cells (NSCs), embryonic fibroblasts (MEFs), and cardiomyocyte cells (HL-1) (Figs. 1A, S1A and S1B; Videos S1–5). The total mitochondrial volume in an ESC is similar to that of an EpiLC and significantly smaller than that of a NSC, a MEF, and a HL-1 cell (Fig. S1A). The cellular volume of an ESC is similar to that of an EpiLC and a HL-1 cell, smaller than a MEF but larger than a NSC (Fig. S1B). Consequently, the ratio of mitochondrial volume to whole cell volume in an ESC is significantly smaller than in an EpiLC, a NSC, a MEF, or a HL-1 cell (Fig. S1C). These new findings prompted us to consider the contribution of mitochondria to ATP generation and stemness regulation in ESCs.

**Figure 1.**
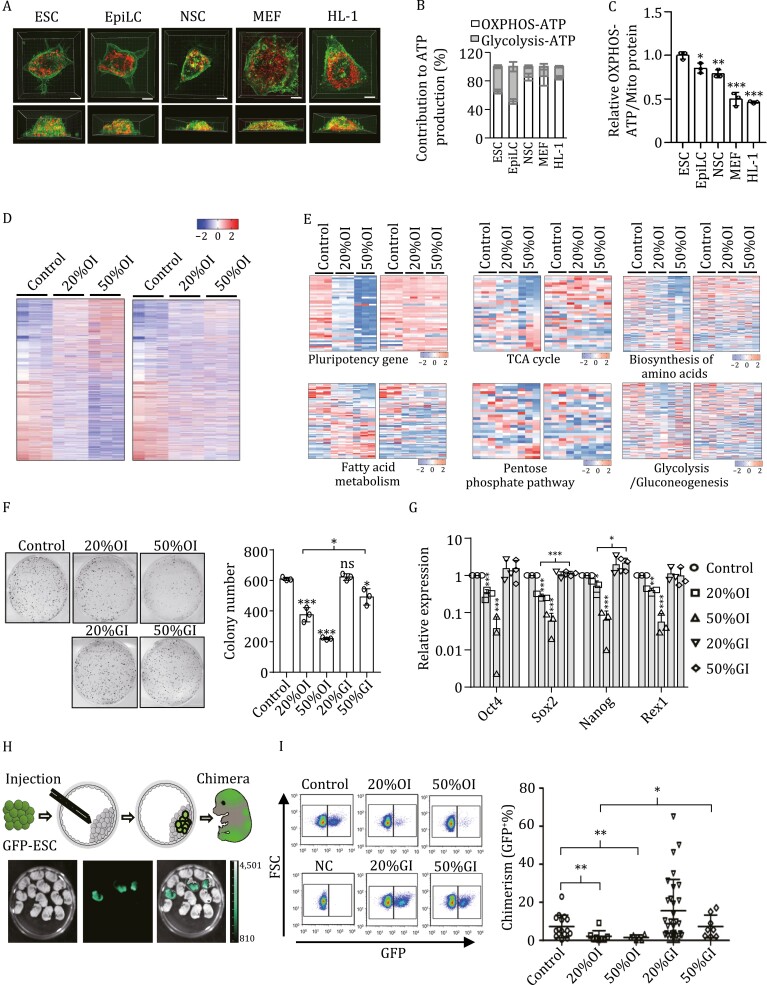
**Super-active mitochondrial respiration in mESCs generates the majority of cellular ATP.** (A) Representative SIM images of a naïve ESC (ESC), a primed ESC (EpiLC), a neural stem cell (NSC), an embryonic fibroblast (MEF), and a cardiomyocyte cell (HL-1). The mitochondria are labeled by mCherry (red) and the cell membranes are stained by DiO (green). Bars, 5 μm. (B) The relative contribution of OXPHOS and glycolysis to ATP production in ESCs (65.16% ± 2.84% vs. 34.84% ± 2.19%), EpiLCs (51.24% ± 3.42% vs. 48.76% ± 6.72%), NSCs (85.41% ± 4.86% vs. 14.59% ± 2.05%), MEFs (86.62% ± 13.24% vs. 13.38% ± 4.00%)), and HL-1 cells (84.49% ± 2.59% vs. 15.51% ± 1.25%). (C) The OXPHOS–ATP generation (OXPHOS-ATP/Mito mass) is significantly higher in ESCs than in EpiLCs, NSCs, MEFs or HL-1 cells. Values are normalized to mitochondrial protein mass. Results are shown as mean ± SD of one representative from three independent experiments. *n* = 3; *P < 0.05; **P < 0.01; ***P < 0.001; Student’s *t*-test. (D) RNA-seq meta-analysis reveals that OXPHOS inhibition results in dramatic changes of gene expression at the whole-transcriptome level. Values displayed correspond to the expression level in the indicated sample scaled by the mean expression of each gene across samples. (E) Inhibition of OXPHOS reprograms expression of pluripotency and metabolic genes. Heatmaps show relative expression levels of genes involved in pluripotency, TCA pathway, amino acid biosynthesis, fatty acid metabolism, pentose phosphate pathway, and glycolysis/gluconeogenesis upon OXPHOS or glycolysis inhibition. (F) Moderate inhibition of OXPHOS, but not glycolysis, diminishes the capacity of ESCs for self-renewal. Left, representative images of alkaline phosphatase staining of colonies formed by ESCs treated with either oligomycin or 2-DG. Right, statistical analysis of the number of alkaline phosphatase-positive colonies. Results are shown as mean ± SD from three independent experiments. *P < 0.05; ***P < 0.001; ns, not significant; Student’s *t*-test. (G) Inhibition of OXPHOS but not glycolysis decreases mRNA expression of pluripotency genes. Results are shown as mean ± SD from three independent experiments. *P < 0.05; **P < 0.01; ***P < 0.001; Student’s *t*-test. (H) Diagram of the chimeric mouse formation assay. Top, after different treatments, GFP-labeled B6 ESCs were injected into CF1 mouse blastocysts, and the blastocysts were transplanted into surrogate mice. Then the chimeric embryos were isolated and digested into single cells at embryonic day 13.5 (E13.5) for FACS analysis. Bottom, representative images of the chimeric embryos isolated from a surrogate mouse at E13.5. (I) Inhibition of OXPHOS, but not glycolysis, decreases the contribution of ESCs to chimeras. Left, GFP-positive cells detected by FACS indicate the number of cells in each chimeric embryo that were derived from the transplanted original cells. Right, summary of data from chimeric embryos. Each dot represents the percentage of GFP^+^ cells in an individual chimeric embryo. Control, *n* = 16; 20%OI, *n* = 8; 50%OI, *n* = 6; 20%GI, *n* = 30; 50%GI, *n* = 8; **P < 0.01; Student’s *t*-test.

The oxygen consumption rate (OCR) and extracellular acidification rate (ECAR) were simultaneously measured in ESCs, EpiLCs, NSCs, MEFs, and HL-1 cells (Fig. S1D and S1E). The absolute quantification of both the oligomycin-sensitive oxygen consumption rate and the glycolytic rate was converted into ATP production rates. Compared to ESCs, EpiLCs and NSCs consumed less oxygen while MEFs and HL-1 cells consumed more oxygen when normalized to equal cell numbers (Fig. S1D). Meanwhile, EpiLCs had a higher glycolytic rate than ESCs, and NSCs, MEFs, and HL-1 cells had a lower glycolytic rate than ESCs (Fig. S1E). Most strikingly however, OXPHOS generates significantly more ATP than glycolysis in an ESC, a NSC, a MEF, and a HL-1 cell. In an EpiLC, OXPHOS and glycolysis generate equal quantities of cellular ATP (Figs. 1B and S1F). Similar results were observed in different ESC or iPSC lines (Fig. S2A and S2D). When ESCs were cultured in 2i medium, the contribution of OXPHOS to ATP generation further increased (Fig. S2B and S2C)

Interestingly, when the OCR was normalized to mitochondrial volume, the ESC mitochondria consumed significantly more oxygen than mitochondria in EpiLCs, NSCs, MEFs, and even HL-1 cardiomyocytes for ATP-generation-related respiration (Fig. S1G). Correspondingly, ESC mitochondria showed a significantly higher ATP generation capacity than mitochondria in EpiLCs, NSCs, MEFs, and HL-1 cells (Fig. S1H). To further strengthen this conclusion, mitochondrial mass was used to normalize ATP-generation-related respiration at the same time. The expression of the mitochondrial protein UQCRC2 was used for normalization, as its quantity per microgram of mitochondrial protein was very similar in each tested line, in contrast to other mitochondrial proteins such as TOM40, TIM23, and ATP5A (Fig. S1I). For each cell line, the mass of an individual cell was determined and UQCRC2 was used for mitochondrial normalization (Fig. S1J–M). ESC mitochondria showed the highest ATP generation capacity among all tested cell lines (Fig. 1C). In addition, the mitochondrial respiration capacity was determined using cells treated with digitonin and the resultant data were normalized to either mitochondrial volume or mitochondrial mass. Using either normalization parameter, ESC mitochondria showed the biggest ATP generation capacity compared to mitochondria in EpiLCs, NSCs, MEFs, and HL-1 cells (Fig. S1N–Q).

To investigate how OXPHOS functions in ESCs, we established assays for dose-dependent inhibition of OXPHOS or glycolysis based on their ATP generation levels. Oligomycin was titrated to inhibit 20% and 50% of the total OXPHOS–ATP generation (designated 20%OI and 50%OI) (Fig. S3A). Meanwhile, 2-deoxy-d-glucose (2-DG), a glucose analog, was titrated to inhibit 20% and 50% of the total glycolysis–ATP generation (designated 20%GI and 50%GI) (Fig. S3B). It is worth mentioning that 20%OI and 50%GI have similar inhibition effects on total cellular ATP generation (Fig. S3C).

Transcriptome profiling was employed to investigate gene expression reprogramming in response to OXPHOS or glycolysis inhibition using the titrated concentrations of oligomycin and 2-DG. Surprisingly, the results indicate that OXPHOS inhibition in ESCs results in much more extensive effects on gene expression than glycolysis inhibition at the whole transcriptome level (Figs. 1D, S4A and S4B; Table S1).

Inhibition of OXPHOS not only decreased expression of pluripotency genes, but also disrupted expression of genes in the tricarboxylic acid (TCA) cycle, the amino acid biosynthesis, fatty acid metabolism, and pentose phosphate pathways as well as the glycolysis/gluconeogenesis pathways in ESCs (Fig. 1E). The ranked enrichment of GO terms showed more dramatically enhanced clustering of metabolic processes in oligomycin- than 2-DG-treated ESCs (Fig. S4C). The expression of the pluripotency genes is significantly decreased upon inhibition of both OXPHOS and glycolysis (Fig. S4D).

Both 20%OI and 50%OI inhibition significantly decreased ESC colony formation and expression of pluripotency genes, whereas 20%GI did not affect ESC self-renewal and pluripotency, and 50%GI inhibited ESC self-renewal and pluripotency to a lesser extent than 20%OI (Figs. 1F, 1G, S5A and S5B). Importantly, the decreased levels of colony formation resulting from OXPHOS inhibition were partially recovered when oligomycin was withdrawn for different lengths of time (Fig. S5C). In addition, treatment with oligomycin or 2-DG at the titrated concentrations for 20%OI, 50%OI, 20%GI, and 50%GI did not enhance ESC apoptosis (Fig. S3E).

ESCs treated with 20%OI or 50%OI did not form any visible teratomas, while 20%GI or 50%GI had no obvious effects on teratoma formation (Fig. S5D–G). Accordingly, the chimerism rate with 20%OI or 50%OI ESC was significantly lower that with mock-treated ESCs. In contrast, neither 20%GI nor 50%GI decreased the chimerism rate (Fig. 1H and 1I).

Consistent with the chemical treatment results, the mitochondrial respiration, self-renewal, pluripotency, and differentiation capability of ESCs were inhibited by ATP5a1 knockdown (Figs. S6 and S7). These data confirmed the function of OXPHOS in safeguarding ESC identity.

Considering that OXPHOS inhibition reprograms metabolic gene expression, we asked whether the disruption of ESC self-renewal and pluripotency is attributed to defective metabolite-mediated signal transduction. To this end, targeted profiling of metabolites was performed to detect the metabolites that changed in response to OXPHOS inhibition. Interestingly, UDP-*N*-acetylglucosamine (UDP-GlcNAc), an amino sugar produced by the hexosamine biosynthetic pathway (HBP), was identified at the top of the metabolite list with a dramatic reduction upon inhibition of OXPHOS, and the expression levels of enzymes involved in UDP-GlcNAc biosynthesis were dramatically disturbed (Figs. 2A and S8A; Table S2).

**Figure 2.**
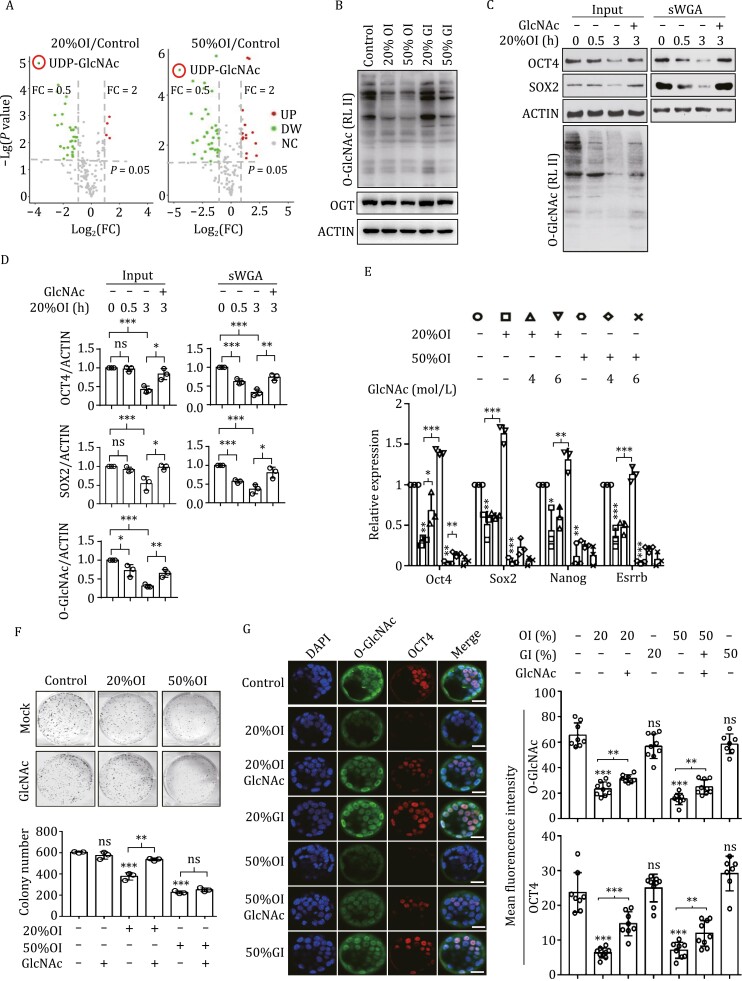
**OXPHOS couples with UDP-GlcNAc generation to safeguard pluripotency.** (A) Visualization of differential metabolite profiles by Volcano plots. UDP-GlcNAc (circled in red) is the top down-regulated metabolite upon OXPHOS inhibition. Red dots represent up-regulated metabolites, FC ≥ 2, *P* ≤ 0.05; green dots represent down-regulated metabolites, FC ≤ 1/2, *P* ≤ 0.05. *P* = 0.05 is labeled by the dotted line. FC, fold change. (B) OXPHOS inhibition results in decreased O-GlcNAcylation of cellular proteins independent of OGT expression. O-GlcNAcylation of total cellular protein was detected by western blotting using an anti-O-GlcNAc antibody. Images are representative of three independent experiments. (C) OXPHOS inhibition causes decreased O-GlcNAcylation and expression of SOX2 and OCT4. These effects are ameliorated by GlcNAc. Decrease of SOX2/OCT4 O-GlcNAcylation initiates 0.5 h after oligomycin addition which is before SOX2/OCT4 downregulation. Cell lysates from ESCs, ESCs treated with oligomycin, or ESCs treated with oligomycin plus GlcNAc for different lengths of time were pulled down by sWGA-agarose and blotted with anti-OCT4, SOX2, O-GlcNAc and β-ACTIN antibodies. (D) Statistical analysis of the western blot results shown in C. *n* = 3; *P < 0.05; **P < 0.01; ***P < 0.001; ns, not significant; Student’s *t*-test. (E) OXPHOS inhibition causes decreased transcription of pluripotency genes, which is partially ameliorated by the addition of GlcNAc. Results are shown as mean ± SD from three independent experiments. *P < 0.05; **P < 0.01; ***P < 0.001; Student’s *t*-test. (F) OXPHOS inhibition reduces the colony formation capability of ESCs. This effect is partially ameliorated by the addition of GlcNAc. Top, representative images of ESC colonies stained by alkaline phosphatase; bottom, statistical analysis of alkaline phosphatase-positive colonies. Results are shown as mean ± SD from three independent experiments. **P < 0.01; ***P < 0.001; ns, not significant; Student’s *t*-test. (G) Decreased O-GlcNAcylation and expression of OCT4 by OXPHOS inhibition in the inner cell mass of *ex vivo*-cultured blastocysts are recovered by addition of GlcNAc. Left, immuno-detection was performed with antibodies specific to O-GlcNAc (green) and OCT4 (red), nuclei were stained with DAPI, Bars, 25 µm; right, statistical analysis of the mean fluorescence intensity. Control, *n* = 8; 20%OI, *n* = 9; 20%OI + GlcNAC, *n* = 8; 20%GI, *n* = 9; 50%OI, *n* = 8; 50%OI + GlcNAC, *n* = 9; 50%GI, *n* = 7; **P < 0.01; ***P < 0.001; ns, not significant; Student’s *t*-test.

UDP-GlcNAc serves as the substrate for O-GlcNAcylation of critical regulators of diverse cellular processes, including several known pluripotency factors (Myers et al., 2011). O-GlcNAcylation ensures pluripotency in ESCs by directly regulating the transcriptional activities of key components of the pluripotency network (Jang et al., 2012). The upstream signals that regulate O-GlcNAcylation in ESCs are unknown. Intriguingly, inhibition of OXPHOS led to a dramatic decrease of UDP-GlcNAc and global O-GlcNAcylation, including O-GlcNAcylation of SOX2/OCT4, without affecting O-linked β-d-*N*-acetylglucosamine transferase (OGT) expression (Figs. 2B–D, S8B and S8C). The decrease of SOX2/OCT4 O-GlcNAcylation began as early as 0.5 h after oligomycin addition, prior to downregulation of SOX2/OCT4 expression (Figs. 2C, 2D, S8B and S8C). In contrast, glycolysis inhibition had little effect (Fig. 2B). In addition, inhibition of UDP-GlcNAc biosynthesis with 6-diazo-5-oxo-l-norleucine (Don) inhibited colony formation and expression of pluripotency genes in ESCs (Fig. S8D). Thus, UDP-GlcNAc links OXPHOS to pluripotency.

To further clarify that OXPHOS regulates ESC identity through UDP-GlcNAc, we added GlcNAc or glucosamine to oligomycin-treated ESCs to rescue the deterioration in ESC identity. As expected, global protein O-GlcNAcylation as well as the expression of OCT4 and SOX2 were partially restored by adding GlcNAc or glucosamine into OXPHOS-inhibited ESCs (Figs. 2C, 2D, S8B, S8C and S8E). Correspondingly, the reduced expression of pluripotency genes, the decreased colony formation capacity, and deficiency of chimera contribution in OXPHOS-inhibited ESCs were also compensated by adding GlcNAc (Figs. 2E, 2F, S8F and S8K). OXPHOS inhibition significantly impaired the proliferation capacity of ESCs, which cannot be rescued by adding GlcNAc (Fig. S8J). Adding GlcNAc into OXPHOS-inhibited ESCs partially restored the gene expression pattern at the whole transcriptome level as well (Fig. S8G). Together, these data support the idea that OXPHOS maintains pluripotency through UDP-GlcNAc.

Importantly, inhibition of OXPHOS, but not glycolysis, resulted in decreased O-GlcNAcylation and expression of Oct4 and Sox2 in the inner cell mass (ICM, the *in vivo* equivalent of ESCs) of blastocysts (Figs. 2G, S8H and S8I). The deterioration in pluripotency gene expression in the ICM upon OXPHOS inhibition can be partially rescued by directly supplementing GlcNAc. Thus OXPHOS safeguards pluripotency via UDP-GlcNAc *in vivo*.

As PSCs undergo differentiation, many cellular parameters change, like cell volume, cell mass, mitochondrial volume, mitochondrial mass, and expression of the mitochondrial house-keeping genes TOM40, TIM23, ATP5A, etc. (Fig. S1A, S1B, S1I and S1K). These changes make it difficult to objectively compare the mitochondrial state in cells at distinct developmental stages. Taking advantage of advanced resolution structured illumination microscopy technology, we determined the absolute volume of individual cells and their mitochondria. The volume of mitochondria in a naïve ESC is significantly lower than that in an NSC, an MEF, or an HL-1 cardiomyocyte, and is similar to that in an EpiLC. In terms of cell volume, an ESC is similar to an EPiLC or an HL-1 cell, significantly smaller than an MEF and significantly larger than an NSC (Figs. 1A, S1A and S1B; Movies 1–5). Consequently, although naïve PSCs consume less oxygen than MEFs and HL-1 cells, and consume more oxygen than EpiLCs and NSCs for ATP generation when normalized to cell number, naïve ESC mitochondria consume significantly more oxygen for ATP generation than mitochondria in primed EpiLCs, somatic stem cells (NSCs), somatic fibroblasts, and HL-1 cardiomyocytes, when normalized to mitochondrial volume (Fig. S1D, S1F–H, S1O and S1P). Mitochondria in naïve ESCs show the largest OXPHOS–ATP generation capacity among the tested cell lines when normalized to mitochondrial protein mass (Figs. 1C and S1Q). These results reveal the functionality of mitochondria in PSCs at distinct pluripotent states and somatic cells at different developmental stages, and partially explain the paradoxical opinions derived from the existing data in the literature. Our data support the hypothesis that naïve PSC mitochondria are more active than mitochondria from PSC progeny when normalized to mitochondrial volume or mass.

We established that OXPHOS accounts for ~65% of total cellular ATP generation in naïve ESCs and ~51% of total cellular ATP generation in primed ESCs (Figs. 1B and S1F). These data led us to conclude that OXPHOS rather than glycolysis generates the majority of the cellular ATP in naïve ESCs, while OXPHOS generates a similar level of ATP as glycolysis in primed EpiLCs.

Both diffusion map and scatterplots analyses indicate that OXPHOS inhibition induces ESCs into a unique state that is different from either the diapause or primed state (Fig. S9). Our integrated transcriptome and metabolome analysis revealed that OXPHOS inhibition causes incomplete catabolism of glucose and abnormal metabolism of nucleotides, glutamine, and acetyl-CoA, thus significantly decreasing the cellular UDP-GlcNAc level in ESCs (Fig. S10). Together, these data suggest that the cellular UDP-GlcNAc concentration is tightly regulated by OXPHOS, and that UDP-GlcNAc serves as a critical cell fate regulator in PSCs.

In conclusion, the current study demonstrates that PSC mitochondria are in a super-active state and OXPHOS produces the majority of cellular ATP, which challenges the traditional concept that ESCs rely on glycolysis as their major source of energy. In addition, this study uncovered a previously unknown mechanism in which OXPHOS couples with the HBP pathway for UDP-GlcNAc generation to regulate pluripotency in mouse ESCs (Fig. S11). The underlying mechanisms by which ESC mitochondria maintain a high respiration rate and favor UDP-GlcNAc production for pluripotency regulation remain to be further investigated.

## Supplementary Material

pwac009_suppl_Supplementary_Material

pwac009_suppl_Supplementary_Table_S1

pwac009_suppl_Supplementary_Table_S2

pwac009_suppl_Supplementary_Video_S1

pwac009_suppl_Supplementary_Video_S2

pwac009_suppl_Supplementary_Video_S3

pwac009_suppl_Supplementary_Video_S4

pwac009_suppl_Supplementary_Video_S5

## Footnotes

We thank Dr. Dangsheng Li from *Cell Research*, Dr. Shyh-Chang, N. from IOZ-CAS, Dr. Jing Yang at Peking University and Drs. Suneng Fu and Maojun Yang from Tsinghua University for their constructive discussions and suggestions. We thank Dr Masaru Okabe at Osaka University Japan for providing the B6D2-Tg (CAG/Su9-DsRed2, Acr3-EGFP) RBGS002Osb mice (mito-red). We thank Dr Noboru Mizushima at the National Institute for Basic Biology Japan for providing the B6.Cg-Tg (CAG-GFP/LC3) 53Nmz/NmzRbrc mice. We thank Yun Feng from the Center for Biological Imaging (CBI), Institute of Biophysics, Chinese Academy of Science for help with SIM image analysis.

Jiani Cao and Tongbiao Zhao designed the experiments, analyzed the data, and wrote the paper; Jiani Cao, Meng Li, Kun Liu, Xingxing Shi, Ning Sui, Yuchen Yao, Xiaojing Wang, Shiyu Li, Yuchang Tian, Qian Zhao, Liang Wang, Xiahua Chai, Lin Zhang, and Xing Li performed the experiments under the overall coordination of Tongbiao Zhao; Chong Liu and Dong Li contributed to the measurement of the volume of mitochondria and cells; Meng Li, Shaojing Tan, and Zhijie Chang analyzed transcriptome and metabolomics data.

J.C., M.L., K.L., X.S., N.S., Y.Y., X.W., S.L., Y.T., S.T., Q.Z., L.W., X.C., L.Z., C.L., X.L., Z.C., D.L., T.Z. declare they have no conflicts of interests. All institutional and national guidelines for the care and use of laboratory animals were followed.

This work was supported by grants from the National Key R&D Program of China 2018YFA0108402, the Strategic Priority Research Program of the Chinese Academy of Sciences XDA16030302, the Strategic Collaborative Research Program of the Ferring Institute of Reproductive Medicine Grant No.33, and the National Natural Science Foundation of China Program (31720103907, 31570995, 31621004), to T.Z. and (31400831) to J.C.

The raw data FASTQ files for the RNA sequencing data are deposited in NCBI GEO, GEO: GSE140712 (Fig. 1D, 1E and S4) and ScienceDB, China National Standard GB/T 7714-2015 (Fig. S8A, S8F and S8G. The metabolomic data files are deposited in ScienceDB, China National Standard GB/T 7714-2015. All the relevant data supporting the findings of this study are also available from the lead contact (tbzhao@ioz.ac.cn) upon request.
