# Supplementary material for: Oxidative phosphorylation safeguards pluripotency via UDP-N-acetylglucosamine
Source: Protein Cell. 2022 Oct 14;14(5):376–81. doi: 10.1093/procel/pwac009 (PMC10166152; doi:10.1093/procel/pwac009)
Supplement: pwac009_suppl_Supplementary_Material [file pwac009_suppl_supplementary_material.pdf]

Oxidative phosphorylation safeguards pluripotency via UDP-N-acetylglucosamine

Supplemental information

Materials and Methods

Figure legends S1 – S11

Movie S1-ESC SIM Image

Movie S2-EpiLC SIM Image

Movie S3-NSC SIM Image

Movie S4-MEF SIM Image

Movie S5-HL1 SIM Image

Table S1

Table S2

Materials and Methods

Key resources table

REAGENT or RESOURCE	SOURCE	IDENTIFIER
Antibodies		
Rabbit polyclonal OCT4 antibody	Abcam	Cat#ab19857; RRID:AB_445175
Mouse monoclonal OCT4 antibody	Santa cruz	Cat#sc-5279; RRID:AB_628051
Rabbit polyclonal SOX2 antibody	Millipore	Cat#AB5603; RRID:AB_2286686
Rabbit polyclonal SOX2 antibody	Abcam	Cat#ab97959; RRID:AB_2341193
Rabbit polyclonal Nanog antibody	Abcam	Cat#ab80892; RRID:AB_2150114
Mouse monoclonal ATP5A antibody	Abcam	Cat# ab176569, RRID:AB_2801536
Mouse monoclonal UQCRC2 antibody	Abcam	Cat# ab14745, RRID:AB_2213640
Rabbit polyclonal TOM 40 antibody	Fine Test	Cat# FNab08860
Rabbit polyclonal TIM 23 antibody	Fine Test	Cat# FNab08693
Mouse monoclonal [RL2] to O-Linked N-Acetylglucosamine antibody	Abcam	Cat#ab2739; RRID:AB_303264
Rabbit monoclonal [EPR12713] to OGT / O-Linked N-Acetylglucosamine Transferase	Abcam	Cat#ab177941
Mouse Monoclonal Anti-β-Actin antibody	Sigma	Cat#A5441; RRID:AB_476744
anti-mouse IgG (H+L), F(ab') 2 Fragment (Alexa Fluor 488 Conjugate)	Cell Signaling Technology	Cat#4408; RRID:AB_10694704
anti-rabbit IgG (H+L), F(ab') 2 Fragment (Alexa Fluor 647 Conjugate)	Cell Signaling Technology	Cat#4414; RRID:AB_10693544
HRP-conjugated goat anti-rabbit IgG	Beyotime	Cat#A0208
HRP-conjugated goat anti-mouse IgG	Beyotime	Cat#A0216
Bacterial and Virus Strains		
Tet-pLKO-puro lentiviral vector	Wiederschain et al., Cell Cycle. 2009 Feb 1. 8(3):498-504	Addgene: 21915; RRID:Addgene_21915
pSPAX2	Didier Trono	Addgene: 12260; RRID:Addgene_12260
pMD2G	Didier Trono	Addgene: 12259; RRID:Addgene_12259

Chemicals, Peptides, and Recombinant Proteins		
Oligomycin	Sigma	Cat#O4876
FCCP	Sigma	Cat#C2920
Rotenone	Sigma	Cat#R8875
Antimycin A	Abcam	Cat#ab141904
2-DG	Sigma	Cat#D8375
Digitonin	Sigma	Cat#D141
Pyruvate	Sigma	Cat#107360
Malic acid	Sigma	Cat#02288
Adenosine 5′-diphosphate sodium salt	Sigma	Cat#A2754
6-Diazo-5-oxo-L-norleucine	Selleckchem	Cat#S8620
TMRE	Thermos Fisher	Cat#T669
Deep Red FM	Invitrogen	Cat#M22426
Glucosamine hydrochloride	Sigma	Cat#G1514
N-Acetyl-D-glucosamine	Sigma	Cat#A3286
Puromycin	Gibco	Cat#A1113802
Doxycycline	Sigma	Cat#D9891
DiO	Yeasen	Cat#40725ES10
BSA(Fatty Acid & IgG Free)	Beyotime	Cat#ST025
$MgCl_2$	Sigma	Cat#M2393
KH_2PO_4	Sigma	Cat#P5655
HEPES	Sigma	Cat#H3375
EGTA	Sigma	Cat#E3889
Sucrose	Sigma	Cat#S7903
D-Mannitol	Sigma	Cat#M4125
Agarose Wheat Germ Agglutinin (WGA), Succinylated	Vector Laboratories	Cat#AL-1023S
Critical Commercial Assays		
Alkaline Phosphatase Assay Kit	Beyotime	Cat#P0321
Mitochondria Isolation Kit for Cultured Cells	Thermos Fisher	Cat#89874
Luminescent ATP Detection Kit	Promega	Cat#ab113849
RNeasy Mini Kit	Qiagen	Cat#74134
GoTaq® qPCR Master Mix	Promega	Cat#PRA6101
SuperScript™ III First-Strand Synthesis System	Invitrogen	Cat#18080051
Deposited Data		
Raw and analyzed data	This paper	GEO: GSE140712
Experimental Models: Cell Lines		
Mouse: B6 ESC	This paper	N/A
Mouse: 129 ESC	This paper	N/A
Mouse: mito-red ESC	This paper	N/A
Mouse: GFP ESC	This paper	N/A
Mouse: Tet-shATP5a1	This paper	N/A
Mouse: Tet-scr	This paper	N/A
Mouse: B6 MEF	This paper	N/A
Mouse: 129MEF	This paper	N/A
Experimental Models: Organisms/Strains		

Mouse: C57BL/6J	Jackson Laboratory	IMSR Cat# JAX: 000664; RRID:IMSR_JAX:000664
Mouse: 129X1/SvJ	Jackson Laboratory	IMSR Cat# JAX: 000691; RRID:IMSR_JAX: 000691
Mouse: B6D2-Tg(CAG/Su9-DsRed2,Acr3-EGFP)RBGS002Osb	Riken	IMSR Cat# RBRC03743; RRID:IMSR_RBRC03743
Mouse: B6.Cg-Tg(CAG-GFP/LC3)53Nmz/NmzRbrc	Riken	IMSR Cat# RBRC00806; RRID:IMSR_RBRC00806
Mouse: Nude mice	Charles River	NU/NU
Mouse: CF1 mice	SLACCAS	CF1
Oligonucleotides		
shRNA targeting ATP5a1 : 5'-TGCAGCCAAGATGAACGATTCCTTCAAGAGAGGAATCGTTCATCTTGGCTGCTTTTTTC-3’	This paper	N/A
scramble shRNA : 5'-TGCTTACGCTGAGTACTTCGAATTCAAGAGA TTCGAAGTACTCAGCGTAAGCTTTTTTC-3’)	This paper	N/A
Primers for real-time PCR, see Materials and Methods	This paper	N/A
Recombinant DNA		
p-Tet-scr	This paper	N/A
p-Tet-shATP5a1	This paper	N/A
pCDH-CAG- TetR-ires- mcherry	This paper	N/A
pCDH-CAG-mcherry	This paper	N/A
Software and Algorithms		
Prism 8	GraphPad Software	https://www.graphpad.com/scientific-software/prism/
Cytoscape	ShannonP et al., 2013	https://cytoscape.org/
Imaris 9.2.1	OXFORD Instruments	https://imaris.oxinst.com
Rstudio	Allaire et al., 2012	https://rstudio.com/
ImageGP	EHBIO gene technology	http://www.ehbio.com/ImageGP/
ImageJ	National Institutes of Health	https://imagej.nih.gov/ij/
Other		

Reagents and Antibodies

Reagents: Oligomycin (Sigma, O4876), FCCP (Sigma, C2920), rotenone (Sigma, R8875), antimycin A (Abcam, ab141904), 2-DG (Sigma, D8375), GlcNAc (Sigma, A3286), puromycin (Gibco, A1113802), doxycycline (Dox; Sigma, D9891), glucosamine (Sigma, G1514), 6-diazo-5-oxo-L-norleucine (Selleckchem, S8620), digitonin (Sigma, #D141), ADP (Sigma, A2754), pyruvate (Sigma, 107360), malic acid (Sigma, 02288), EGTA (Sigma, E3889), HEPES (Sigma, H3375), sucrose (Sigma, S7903), D-mannitol (Sigma, M4125), $MgCl_2$ (Sigma, M2393), KH_2PO_4 (Sigma, P5655), and BSA (Fatty Acid & IgG Free) (Beyotime, ST025) were used for cell treatments and Seahorse XF24 extracellular flux analysis. For extended resolution structured illumination imaging (SIM), DiO (Yeasen, 40725ES10) was used to stain cell membranes, Deep Red FM (Invitrogen, M22426) was used to stain mitochondria in EpiLCs and HL-1 cells, and TMRE (Thermos Fisher, T669) was used to stain mitochondria in NSCs. Mitochondria in ESCs and MEFs were labeled by mCherry.

Antibodies: anti-OCT4 (Abcam, ab19857, 1:1000 for western blot (WB); 1:100 for immunofluorescence analysis (IFA) of mESC), anti-OCT4 (Santa Cruz, sc-5279, 1:100 for IFA of blastocysts), anti-SOX2 (Millipore, AB5603, 1:100 for IFA of blastocysts), anti-SOX2 (Abcam, ab97959, 1:1000 for WB), anti-NANOG (abcam, ab80892, 1:1000 for WB), anti-O-linked N-acetylglucosamine (Abcam, ab2739, 1:1000 for WB, 1:50 for IFA of blastocysts), anti-OGT (Abcam, ab177941, 1:1000 for WB), anti-ATP5A (Abcam, ab176569, 1:1000 for WB), anti-UQCRC2 (Abcam, ab14745, 1:1000 for WB), anti-TOM 40 (Fine Test, FNab08860, 1:1000 for WB), anti-TIM 23 (Fine Test, FNab08693, 1:1000 for WB) and anti-β-ACTIN (A5441, Sigma, 1:1000 for WB) were used as primary antibodies in western blotting and immunofluorescence

imaging assays; anti-mouse IgG (H+L), F(ab')$_2$ Fragment (Alexa Fluor 488 Conjugate) (CST, 4408, 1:1000) and anti-rabbit IgG (H+L), F(ab')$_2$ Fragment （Alexa Fluor 647 Conjugate）(CST, 4414, 1:1000) were used as secondary antibodies for immunofluorescence; HRP-conjugated goat anti-rabbit (A0208, Beyotime, 1:10000) and goat anti-mouse (A0216, Beyotime,1:10000) IgG were used as secondary antibodies for western blotting.

Plasmids and Lentivirus

The shRNA targeting ATP5a1 (5'-TGCAGCCAAGATGAACGATTCCTTCAAGAGAGGAATCGTTCATCTTGGCTGCTTTTTTC-3') and the scramble shRNA (5'-TGCTTACGCTGAGTACTTCGAATTCAA GAGA TTCGAAGTACTCAGCGTAAGCTTTTTTC-3') were cloned into the Tet-pLKO-puro lentiviral vector (a gift from Dmitri Wiederschain, Addgene, 21915), to create constructs named p-Tet-scr and p-Tet-shATP5a1 respectively. A cDNA encoding tetracycline repressor protein (TetR) was cloned into the pCDH-CAG-mcherry lentiviral expression vector, to create the construct named p-TetR. The lentivirus packaging vectors pSPAX2 and pMD2G were gifts from Didier Trono (Addgene, pSPAX, 12260; pMD2G, 12259). To prepare lentivirus, HEK293T cells were transduced with the plasmids p-Tet-scr/pSPAX2/pMD2G, p-Tet-shATP5a1/pSPAX2/pMD2G and p-TetR/pSPAX2/pMD2G using the calcium phosphate-DNA coprecipitation method. Medium containing the virus was collected 48 h after transfection.

Mice

All animal experiments were approved by the Ethics Committee in the Institute of Zoology, Chinese Academy of Sciences in accordance with the Guidelines for Care and Use of Laboratory Animals

established by the Beijing Association for Laboratory Animal Science. C57BL/6 (Jackson Laboratory, 000664), 129X1/SvJ (Jackson Laboratory, 000691), mito-red B6D2-Tg(CAG/Su9-DsRed2,Acr3-EGFP)RBGS002Osb (Riken, RBRC03741) {Hasuwa, 2010 #72} and B6.Cg-Tg(CAG-GFP/LC3)53Nmz/NmzRbrc mice (Riken, RBRC00806) {Mizushima, 2004 #79} were used for preparation of mouse embryonic fibroblasts (MEFs) and establishing ESC lines. C57BL/6 (Jackson Laboratory, 000664) mice were used for preparation of mouse neural stem cells (NSCs). Nude mice (Charles River, NU/NU) were used in teratoma formation assays. CF1 mice (SLACCAS) were used for feeder cell preparation and chimerism assays.

METHOD DETAILS

Generation of Stable ESC Lines

B6 ESCs were infected with lentivirus harboring TetR with the mcherry cassette, and the positive colonies were picked based on the mcherry fluorescence. Then the cells were infected with p-Tet-scr or p-Tet-shATP5a1, and selected with 1 μg/ml puromycin. The surviving colonies were picked and confirmed by sequencing before use in further experiments.

Cell Culture

MEFs were isolated from embryonic day 13.5 (E13.5) fetal mice and passage 3 (P3) MEFs were used in each experiment. NSCs were isolated from cerebral cortex of E13.5 fetal mice. B6 ES (C57BL/6), J1 ES (129X1/SvJ) and Red-mito ES cell lines were isolated from E3.5 blastocysts and cultured on feeder layers for 5 d, and then routinely passaged. 1E iPS and 2E iPS cells were used as previously described {Zhao, 2011 #71}. MEFs and HEK293T cells were maintained in DMEM High Glucose with 10% fetal bovine serum, 2 mM glutamine, 1 mM sodium pyruvate, and 1% penicillin/streptomycin at 37°C in 5% CO_2. ESCs and iPSCs were maintained in KnockOut DMEM

with 15% fetal bovine serum, 2 mM glutamine, 1 mM sodium pyruvate, 0.1 mM nonessential amino acids, 1% penicillin/streptomycin, 0.055 mM β-mercaptoethanol, and 1,000 U/ml leukemia inhibitory factor (Millipore). Specifically, the 2i ESCs were maintained in a 1:1 mix of DMEM/F12 (Gibco, 11302-33) and Neurobasal medium containing N2 supplements, B27 supplements, 10% KSR, 0.2% BSA, 1% penicillin/streptomycin, 0.1 mM β-mercaptoethanol, 2 mM glutamine and LIF supplemented with 1 μM PD03259010 (Stemgent), 3 μM CHIR99021 (Stemgent). The medium was changed every day. After three days of induction, the cells were passaged as cell clumps by dissociating with collagenase IV (1 mg/ml; Invitrogen), and maintained in the medium described as above but supplemented with 20% KSR. NSCs were maintained in Neurobasal medium containing B27 supplements, Glutamax, bFGF (20 ng/ml), EGF (20 ng/ml). HL-1 cells were maintained in MEM medium supplemented with 10% fetal bovine serum and 1% penicillin/streptomycin. EpiLCs were induced by plating 1.5 x 10^6 ESCs on a 10 cm dish coated with human plasma fibronectin (16.7 mg/ml) in the 1:1 mix of DMEM/F12 (Gibco, 11302-33) and Neurobasal medium containing N2 supplements, B27 supplements, activin A (20 ng/ml), bFGF (12 ng/ml), and KSR (1%) {Hayashi, 2011 #96}. All cell culture reagents were purchased from Gibco unless indicated. For 20% or 50% inhibition of OXPHOS ATP production, ESCs were cultured in ESC medium containing 1 nM or 10 nM oligomycin respectively. For 20% or 50% inhibition of glycolysis ATP production, ESCs were cultured in ESC medium containing 5 mM or 10 mM 2-DG respectively. The established Tet-scr and Tet-shATP5a1 ESC lines were cultured in ESC medium containing 0, 125 ng/ml, or 250 ng/ml Dox. For HBP inhibition, ESCs were cultured in ESC medium containing 25 μM 6-diazo-5-oxo-L-norleucine. For the O-GlcNAcylation rescue assay, glucosamine (Sigma, G1514) or GlcNAc (Sigma, A3286) was supplemented as indicated.

Immunofluorescence Microscopy

ESCs cultured on gelatin-coated glass slides were fixed with 4% paraformaldehyde for 30 min, washed with Dulbecco's PBS (Hyclone), permeabilized by 0.2% Triton-X100 (Sigma Aldrich, X100) for 20 min, blocked with 2% bovine serum albumin (Sigma) for 1 h, stained with the indicated primary antibodies for 2 hour at 37°C, and then incubated with corresponding secondary antibodies for 2 h at room temperature.

For SIM, Mito-red ESCs and Mito-red MEFs cultured on gelatin-coated glass slides were fixed with 4% paraformaldehyde for 30 min, washed with Dulbecco's PBS (Hyclone), and stained with 10 μM DiO for 5 min at 37°C; EpiLCs and HL-1 cells cultured on fibronectin-coated glass slides were stained with 20 nM Deep Red FM for 5 min at 37°C and then fixed with 4% paraformaldehyde for 30 min, washed with Dulbecco's PBS (Hyclone), and stained with 10 μM DiO for 5 min at 37°C; NSCs cultured on Matrigel-coated glass slides were stained with 100 nM TMRE for 5 min at 37°C and stained with 10 μM DiO for 5 min at 37°C. Then the samples were visualized by a 3D-SIM built by Dr. Dong Li's group {Wang, 2021 #105}. Briefly, the 3D-SIM was built based on an inverted fluorescence microscope (IX83, Olympus). The excitation light from a laser combiner equipped with 488 nm (500mW, Coherent Genesis Max), and 560 nm (500mW, MPB Communications, VFL-P-500-560) was passed through an acousto-optic tuneable filter (AOTF, AA Quanta Tech), which selected the excitation wavelength and controlled the power, coupled into an afterwards light path. The output beam from the AOTF was expanded to ~15 mm and sent into a phase modulator consisting of a polarizing beam splitter, an achromatic half-wave plate (HWP; Bolder Vision Optik), and a ferroelectric spatial light modulator (SLM; Forth Dimension Displays, QXGA-3DM). The light diffracted by the grating pattern displayed on the SLM was directed to a

polarization rotator (Meadowlark) to adjust the linear polarization of excitation light so as to maintain the s-polarization to maximize the interference pattern contrast. After selecting the 0 and ±1 diffraction orders, the excitation light was relayed into the back pupil of the objective (UPLSAPO100XS, Olympus), after which the collimated excitation light formed the interference pattern to illuminate the specimen. Meanwhile, the fluorescence was collected by the same objective, and relayed to a sCMOS camera (Hamamatsu, Orca Flash 4.0 v3) to acquire the raw images. The z-slices were sequentially acquired with 0.16 μm intervals to form a 3D-SIM image. The exposure time for each raw image was 10 ms at 20 W/cm.

The SIM images were rendered into 3D volumes and analyzed using Imaris V9.2.1. Cell surfaces were extracted using the Surface tools with manual editing, and the 3D structures of mitochondria were extracted by the Surface segmentation utility which detect objects based on local intensities. Images and videos were captured using the snapshot function and the animation function, respectively.

Blastocysts

Two-cell embryos were isolated from E1.5 mouse embryos after the onset of pregnancy, and cultured in KSOM medium (Millipore, MR-121-D) at 37°C in 5% CO_2 for 2 days. Then the blastocysts were randomly dived into 7 groups: Control (normally cultured in KSOM, 37°C, 5% CO_2, 4 hours); 20%OI (cultured in KSOM supplemented with 1 nM oligomycin, 37°C, 5% CO_2, 4 h); 20%OI+GlcNAc (cultured in KSOM supplemented with 1 nM oligomycin and 6 mM GlcNAc, 37°C, 5% CO_2, 4 h), 20%GI (cultured in KSOM supplemented with 5 mM 2-DG, 37°C, 5% CO_2, 4 h), 50%OI (cultured in KSOM supplemented with 10 nM oligomycin, 37°C, 5% CO_2, 4 h); 50%OI+GlcNAc (cultured in KSOM supplemented with 10 nM oligomycin and 6 mM GlcNAc,

37°C, 5% CO_2, 4 h) and 50%GI (cultured in KSOM supplemented with 10 mM 2-DG, 37°C, 5% CO_2, 4 h).

Inner Cell Mass Staining

After receiving the indicated treatments, *ex vivo* blastocysts were fixed with 4% paraformaldehyde for 20 min, washed with Dulbecco's PBS (Hyclone), permeabilized by 0.5% Triton-X100 (Sigma Aldrich, X100) for 1 h, blocked with 2% bovine serum albumin (Sigma) for 1 h, washed with Dulbecco's PBS (Hyclone), stained with the indicated primary antibodies overnight at 4°C and then incubated with corresponding secondary antibodies for 1 h at room temperature. Cell nuclei were counterstained with DAPI.

XF24 Extracellular Flux Analysis

Respiration and acidification rates were measured in cells using a Seahorse XF24 analyzer (Seahorse Bioscience, North Billerica, MA). Briefly, ESCs, EpiLCs, NSCs, MEFs, or HL-1 cells were seeded on 0.1% gelatin-coated XF24 cell culture microplates (Seahorse Bioscience) at 5×10^4 cells/well and incubated at 37°C in 5% CO_2 overnight. For each XF24 extracellular flux assay, two microplates were prepared simultaneously. One microplate was used to measure the metabolism rates while the other one was used to determine the cell number at the time point when the measurement started.

The assays in Fig. 1E-H, Fig. S1A-C,H, Fig. S3A-C and Fig. S6A-B were performed in XF Base Medium (Agilent, 102353-100), supplemented with 25 mM glucose, 2 mM glutamine and 1 mM sodium pyruvate. Cells were washed twice and preincubated in this medium for 1 hour before testing. For ATP production determination, substrates and selective inhibitors were injected during the measurements to achieve final concentrations as following: port A: oligomycin (1 μM), port B:

FCCP (0.3 μM), port C: rotenone (1 μM) and antimycin A (1 μM), port D: 2-DG (100 mM) (Fig.1. E-H, and Fig. S1A-C,H). To titrate the effects of oligomycin/2-DG on ATP production, inhibitors were injected during the measurements to achieve a series of final concentrations of oligomycin (1 nM, 5 nM, 10 nM, 100 nM, 200 nM, and 1000 μM), and 2-DG (2.5 mM, 5 mM, 10 mM and 100 mM) (Fig.S3A-C). For the mitochondrial stress test, inhibitors were injected to achieve final concentrations as following: port A: oligomycin (1 μM), port B: FCCP (0.3 μM), port C: rotenone (1 μM) and antimycin A (1 μM) (Fig.S6A-B). The oxygen consumption rate (OCR), extracellular acidification rate (ECAR) and protein production rate (PPR) values were further normalized to the number of cells present in each well.

The assays in Fig.S1I-L were performed in mannitol and sucrose (MAS) buffer (70 mM sucrose, 220 mM mannitol, 10 mM KH_2PO_4, 5 mM $MgCl_2$, 2 mM HEPES and 1 mM EGTA, 4 mg/ml BSA; pH adjusted the to 7.2 with 0.1 M KOH). Substrates and selective inhibitors were injected during the measurements to achieve final concentrations as follows: port A: digitonin (25 μg/mL), pyruvate (5 mM), malate (2.5 mM) and ADP (1 mM); port B: oligomycin (1 μg/mL); port C: rotenone (1 μM) and antimycin A (20 μM) (Fig.1. E-H, and Fig. S1A-C, H) {Salabei, 2014 #106}.

ATP Production Calculation

The OCR, which drives mitochondrial ATP synthesis, can be calculated by addition of oligomycin (ATP synthase inhibitor), according to Equation 1 (E1) below. To convert the ATP-coupled OCR into the mitochondrial ATP production rate, the P/O ratios were estimated considering the stoichiometry of ATP phosphorylated per atom of oxygen consumed. Based on data from experiments on isolated mitochondria and considering the efficiency of the F1-F0 ATP synthase, the maximum theoretical P/O values obtained vary from 2.45 (palmitate oxidation) to 2.86 (full

glycogen oxidation) (Brand, 2005; Mookerjee et al., 2017). A P/O ratio of 2.75 has been validated for calculating the OXPHOS ATP production rate in cells (Romero N et al Quantifying Cellular ATP Production Rate Using Agilent Seahorse XF Technology). Thus, the OXPHOS ATP production rate can be calculated according to Equation 2 (E2).

E1: OCR_{ATP} (pmol O_2/min) = OCR_{basal} (pmol O_2/min) – OCR_{Oligo} (pmol O_2/min)

E2: OXPHOS ATP production rate (pmol ATP/min) = OCR_{ATP} (pmol O_2/min) * 2 (pmol O/pmol O_2) * 2.75 (P/O rate: pmol ATP/pmol O)

Glycolysis converts glucose to lactic acid, accompanied by extrusion of one H^+ (one proton) per lactate according to Equation 3 (E3) below. ECAR data are converted to PPR (proton production rate) based on the buffer capacity of the assay medium. The glycolytic ATP production rate is equivalent to the glycolytic proton production rate ($PPR_{glycolysis}$), which is calculated as basal PPR minus non-glycolytic PPR (PPR_{2DG}). Thus, the glycolytic ATP production rate can be calculated according to Equation 4 (E4).

E3: Glucose + 2 ADP + 2 Pi ➔ 2 Lactate + 2 ATP + 2 H_2O + 2 H^+

E4: Glycolytic ATP production rate (pmol ATP/min) = $PPR_{glycolysis}$ (pmol H^+/min) = PPR_{basal} (pmol H^+/min) - PPR_{2DG} (pmol H^+/min)

Colony Formation Assay and AP Staining

ESCs were seeded at a density of 2000 cells/well in a six-well plate, and cultured for 7 days. The colonies were stained using an Alkaline Phosphatase Assay Kit (Beyotime, P0321), and the colony numbers were analyzed by Image-Pro Plus software.

Mitochondria Isolation

The mitochondria from ESC, EpiLC, NSC, MEF, or HL-1 cell were isolated by a mitochondria

isolation kit for cultured cells (Thermos Fisher, 89874).

Western Blotting

Cells or the isolated mitochondria were lysed in RIPA buffer (50 mM Tris-HCl, pH 7.4, 150 mM NaCl, 0.5% sodium deoxycholate, 1% Nonidet P-40, 5 mM EGTA, 2 mM EDTA, 10 mM NaF) on ice for 30 min. A protease inhibitor cocktail tablet (04693116001, Roche) and 1 mM PMSF (ST506, Beyotime) were added during the lysis process. Cell lysates were subjected to SDS-PAGE and transferred to PVDF membranes (Millipore). Membranes were blocked with 5% nonfat milk for 1 h at room temperature and then probed with the indicated primary antibodies at 4°C overnight, followed by the appropriate HRP-conjugated secondary antibodies for 1 h at room temperature. After washing with TBST 3 times, the blotted membranes were visualized with chemiluminescent kits (Millipore). Densitometric analyses were performed using ImageJ software.

Immunoprecipitation

The total protein of ESCs was extracted using a cell lysis buffer for Western and IP (Beyotime, P0013). Supernatants were isolated by centrifugation at 13000 × rpm for 15 min, and then incubated with sWGA agarose beads for 14 h at 4 °C with rotation (Vector laboratories, AL-1023S). The samples were centrifuged at 2500 × rpm for 5 min at 4 °C and washed five times with lysis buffer. The proteins coupled to beads were dissociated by boiling the samples at 100 °C for 10 min in 20 ul 5 × loading buffer (Beyotime, P0015L,) and then were subjected to western blot.

Real-time PCR

Total RNA was extracted from samples with an RNeasy Mini Kit (Qiagen). About 1-2 μg total RNA was reverse-transcribed into cDNA using the SuperScript™ III First-Strand Synthesis System

(Invitrogen). Qualitative PCR was performed with GoTaq® qPCR Master Mix (Promega) and a 7500 Fast Real-Time PCR System (Applied Biosystems). All samples were analyzed in duplicate and normalized to β-Actin. The primers used were as follows: Oct4: 5'-AGAGGATCACCTTGGGGTACA-3' (forward), 5'- CGAAGCGACAGATGGTGGTC-3' (reverse); Nanog: 5'-TCTTCCTGGTCCCCACAGTTT-3' (forward), 5'-GCAAGAATAGTTCTCGGGATGAA-3' (reverse); Sox2: 5'-GCGGAGTGGAAACTTTTGTCC-3' (forward), 5'-CGGGAAGCGTGTACTTATCCTT-3' (reverse); Esrrb: 5'-CAG GCAAGGATGACAGACG-3' (forward), 5'-GAGACAGCACGAAGGACTGC-3' (reverse); Rex1: 5'- CCCTCGACAGACTGACCCTAA-3' (forward), 5'- TCGGGGCTAATCTCACTTTCAT -3' (reverse); β-Actin: 5'- GGCTGTATTCCCCTCCATCG -3' (forward), 5'-CCAGTTGGTAACAATGCCATGT -3' (reverse).

Teratoma Formation Assay

Approximately 2×10^6 ESCs were subcutaneously injected into 10-week-old female nude mice. Four weeks later, teratomas were disassociated, weighed and fixed with 4% paraformaldehyde, embedded in paraffin, sectioned, and subjected to H&E staining.

Chimerism Assay

After receiving the indicated treatments, the fluorescence-labeled ESCs were injected into blastocysts, then the injected blastocysts were transplanted into surrogate mice. The chimeric embryos (E13.5) were digested into single cells and subjected to FCAS analysis.

Apoptosis Assay

The apoptosis assay was performed using an Alexa Fluor® 488 Annexin V/Dead Cell Apoptosis Kit (Invitrogen) according to manufacturer's instructions. Briefly, cells were washed with PBS and re-

suspended in binding buffer. The cells were incubated with annexin-V-FITC and propidium iodide (PI) for 20 min at RT, and then subsequently analyzed by flow cytometry. Cells that were annexin-V-negative and PI-negative were counted as viable cells.

CFSE staining assay

The ESCs were incubated with 10 μM carboxyfluorescein succinimidyl ester (Sigma) for 10 min at 37°C and receiving the indicated treatments for 48 hour. Proliferation of cells was analyzed by FACS.

ATP content analysis

After receiving the indicated treatments, the cellular ATP contents were determined by a Luminescent ATP Detection Kit (promega) according to manufacturer's instructions.

RNA-seq, Differential Gene Expression Analysis, and Function Enrichment Analysis

Total RNA was extracted from the indicated cell samples with an RNeasy Mini Kit (Qiagen). RNA sequencing was performed by Annoroad Gene Technology Corporation.

DESeq2 software was used for differential gene expression analysis. Briefly, the expression level of each gene per sample was estimated by DESeq2 according to linear regression, and the p-value was calculated with the Wald test. Finally, the p-value was corrected by the Benjamini-Hochberg (BH) method. Genes with $q \leq 0.05$ and $|log2\_ratio| \geq 1$ were identified as differentially expressed genes (DEGs). The genome-wide heatmaps were plotted with GraphPad Prism 8 software.

GO (Gene Ontology, http://geneontology.org/) enrichment analysis of DEGs was implemented by the hypergeometric test, in which the p-value is calculated and adjusted according to the false

discovery rate to give a q-value, and genes in the whole genome are used as the background data. GO terms with $q<0.05$ were considered to be significantly enriched. GO enrichment analysis assigns biological functions to DEGs. The GO enrichment map was plotted with imageGP.

Diffusion map is a nonlinear dimensionality reduction method where DC represents the diffusion coefficient {Lafon, 2006 #107}. The correlation coefficient for omics data between two groups is calculated as R. All analyses were performed using two-tailed Student's *t*-test (unless otherwise specified).

Metabolomics Analysis

The ESCs were cultured in 10 cm dishes under feeder-free conditions (~10^7 cells/dish) and treated as indicated for 48 h. The cells were washed with cold PBS 3 times and then were incubated in 2 ml 80% (vol/vol) methanol (pre-chilled to -80 °C) at -80°C for 1 h. Cell lysate/methanol mixtures were acquired by scraping, then centrifuged at 14000g for 20 min at 4°C. The protein content of the cell pellets was quantified for normalization. The metabolite-containing supernatants were removed to a new 1.5 ml tube and then dried to a pellet in a Speedvac. The metabolite pellet samples were submitted for the following metabolomic analysis. The targeted metabolomic experiment was analyzed by a TSQ Quantiva triple-stage quadrupole mass spectrometer (Thermo, CA). C18-based reverse-phase chromatography was utilized with 10 mM tributylamine, 15 mM acetate in water and 100% methanol as mobile phases A and B respectively. This analysis focused on TCA cycle, glycolysis pathway, pentose phosphate pathway, amino acid metabolism and purine metabolism. A 25 min gradient from 5% to 90% of mobile phase B was used. Positive-negative ion switching mode was performed for data acquisition. The resolution for Q1 and Q3 are both 0.7 FWHM. The source

voltage was 3500 V for positive and 2500 V for negative ion mode. The source parameters are as follows: spray voltage, 3000 V; capillary temperature, 320 °C; heater temperature, 300 °C; sheath gas flow rate, 35; auxiliary gas flow rate, 10. Metabolite identification was based on a Tracefinder search with a home-built database containing about 300 compounds. Rstudio was used for differential metabolomics compounds analysis, and p-values were calculated with the two-tailed distribution *t*-test based on the two-sample isovariance hypothesis.

Integrated Metabolome and Transcriptome Network Analysis

Based on the metabolites and transcripts detected in our experimental setting and the KEGG REACTION, KEGG ENZYME, KEGG COMPOUND, and KEGG GLYCAN databases, we integrated the metabolites and corresponding enzymes into a global network as previously described (Jha et al., 2015; Kanehisa et al., 2012). The network was annotated and plotted by Cytoscape software.

Statistical analysis

Statistical details and number of replicates are shown in the corresponding figure legends. Analyses were performed using SPSS Software. Statistical significance was calculated using unpaired *t* tests between the indicated groups, P-values <0.05 were considered as statistically significant. P-values are indicated by asterisks as follows: *P<0.05, **P<0.01, ***P<0.001.

Figure legends S1 – S11

Figure S1. Determination of the Contribution of Oxidative Phosphorylation and Glycolysis to ATP Generation in Mouse ESCs, EpiLCs, NSCs, MEFs and HL-1 cells

(**A**) The total mitochondrial volume in an ESC (31.05 ± 12.45 μm^3) is significantly smaller than that

in an NSC (49.02 ± 23.46 μm³), an MEF (174 ± 64.68 μm³) or an HL-1 cell (99.03 ± 42.5 μm³), and is similar to that in an EpiLC (37.4 ± 16.08 μm³). The total mitochondrial volume in individual ESCs, EpiLCs, NSCs, MEFs and HL-1 cells was determined by SIM. Results are shown as mean ± SD; ESC, n=29; EpiLC, n=25; NSC, n=19; MEF, n=25; HL-1, n=26; **P < 0.01; ***P < 0.001; ns, not significant; Student's *t*-test. (**B**) An ESC (1633 ± 476.7 μm³), which is similar in volume to an EpiLC (1435 ± 491.7 μm³) and an HL-1 cell (1830 ± 416.8 μm³), is smaller than an MEF (4895 ± 1296 μm³) but is larger than an NSC (932.9 ± 476.3 μm³). The volumes of individual ESCs, EpiLCs, NSCs, MEFs and HL-1 cells were determined by SIM. Data are shown as mean ± SD; ESC, n=29; EpiLC, n=25; NSC, n=19; MEF, n=25; HL-1, n=26; ***P < 0.001; ns, not significant; Student's *t*-test. (**C**) The ratio of total mitochondrial volume to cell volume in ESCs (1.9% ± 0.5%) is smaller than in EpiLCs (2.72% ± 0.98%), NSCs (5.5% ± 1.68%), MEFs (3.5% ± 0.94%) and HL-1 cells (5.36% ± 1.79%). Results are shown as mean ± SD; ESC, n=29; EpiLC, n=25; NSC, n=19; MEF, n=25; HL-1, n=26; ***P < 0.001; Student's *t*-test. (**D**) Left, Oxygen consumption rate (OCR) measured by Seahorse Extracellular Flux Assay; right, oligomycin-sensitive oxygen consumption rates (Oligomycin OCR) in naïve ESCs, EpiLCs, NSCs, MEFs and HL-1 cells. Results are shown as mean ± SD of three independent experiments; **P < 0.01; ***P < 0.001; Student's *t*-test. (**E**) Left, Extracellular acidification rate (ECAR) measured by Seahorse Extracellular Flux Assay; right, glycolytic rate (ECAR) in naïve ESCs, EpiLCs, NSCs, MEFs and HL-1 cells. Results are shown as mean ± SD of three independent experiments; *P < 0.05; **P < 0.01; ***P < 0.001; Student's *t*-test. (**F**) ATP production by oxidative phosphorylation (OXPHOS-ATP) or glycolysis (Glycolysis-ATP) in 10000 naïve ESCs, EpiLCs, NSCs, MEFs and HL-1 cells. The oligomycin-sensitive oxygen consumption rate is converted into the OXPHOS ATP production rate using a P/O ratio of 2.75, and

ATP production by glycolysis is determined by a one-to-one relationship between the generation of protons and ATP. The ratio of proton production rate to ECAR (PPR/ECAR) was determined as 5.198 using the Seahorse XF24 analyzer based on the buffer capacity of the assay medium. Results are shown as mean ± SD of three independent experiments. n=3; ***P < 0.001; ns, not significant; Student's *t*-test. (**G**) The rate of oligomycin-sensitive oxygen consumption (Oligomycin OCR/Mito volume) is significantly higher in ESCs than in EpiLCs, NSCs, MEFs and HL-1 cells. Values are normalized to mitochondrial volume. Results are shown as mean ± SD of one representative from three independent experiments. n=3; ***P < 0.001; Student's *t*-test. (**H**) The OXPHOS-ATP generation (OXPHOS-ATP/Mito volume) is significantly higher in ESCs than in EpiLCs, NSCs, MEFs or HL-1 cells. Values are normalized to mitochondrial volume. Results are shown as mean ± SD of one representative from three independent experiments. n=3; ***P < 0.001; Student's *t*-test.

(**I**) The level of UQCRC2 protein is constant in mitochondria from naïve ESCs, EpiLCs, NSCs, MEFs and HL-1 cells. The data are normalized to equal mitochondrial protein mass. Left, the level of TOM40, TIM23, ATP5A and UQCRC2 were detected by western blotting in 15 μg mitochondrial proteins isolated from naïve ESCs, EpiLCs, NSCs, MEFs and HL-1 cells. Right, results are shown as mean ± SD of three independent experiments. n=3; ***P < 0.001; ns, not significant; Student's *t*-test. (**J**) Titration of cellular protein quantity in naïve ESCs, EpiLCs, NSCs, MEFs and HL-1 cells. (**K**) Absolute protein quantity in an individual naïve ESC, EpiLC, NSC, MEF and HL-1 cell. (**L**) The relative levels of UQCRC2 in an equal mass of total cellular protein. Left, the expression levels of UQCRC2, OCT4, NANOG, NESTIN and ACTIN in 15 μg cellular proteins from naïve ESCs, EpiLCs, NSCs, MEFs and HL-1 cardiomyocytes were detected by western blotting; right, results

are shown as mean ± SD of three independent experiments. n=3; *P < 0.05; Student's *t*-test. (**M**) ATP production by OXPHOS or glycolysis in naïve ESCs, EpiLCs, NSCs, MEFs and HL-1 cells. Data are normalized to an equal mass of cellular protein. Results are shown as mean ± SD of three independent experiments. n=3; ***P < 0.001; ns, not significant; Student's *t*-test. (**N**) OCR was measured by Seahorse Extracellular Flux Assay using digitonin-permeabilized cells fed complex-specific substrates of the preceding complex in the electron transport chain. DIG, digitonin; Pyr, pyruvate; Mal, malic acid; ADP, Adenosine-5'-diphosphate; Oligo, oligomycin; Rot, rotenone. (**O**) State 3 OCR (OCR values measured after injection of DIG/Pyr/Mal/ADP) in naïve ESCs is significantly higher than in EpiLCs and NSCs, and lower than in MEFs and HL-1 cells, when the data are normalized to equal numbers of cells. Results are shown as mean ± SD of three independent experiments. n=3; **P < 0.01; ***P < 0.001; Student's *t*-test. (**P**) State 3 OCR in naïve ESC is significantly higher than that in EpiLCs, NSCs, MEFs or HL-1 cells when the data are normalized to equal mitochondrial volume. Results are shown as mean ± SD of three independent experiments. n=3; *P < 0.05; **P < 0.01; Student's *t*-test. (**Q**) Relative state 3 OCR in naïve ESCs is significantly higher than in EpiLCs, NSCs, MEFs and HL-1 cells when the data are normalized to equal mitochondrial protein mass. Results are shown as mean ± SD of three independent experiments. n=3; *P < 0.05; ***P < 0.001; Student's *t*-test.

Figure S2. ATP generation by OXPHOS and Glycolysis in Different Somatic Cell and Pluripotent Stem Cell Lines

(A) Left, ATP production by OXPHOS and glycolysis in different ESC and iPSC lines cultured in ESC medium suppled with fetal bovine serum; Right, the relative contribution of OXPHOS and glycolysis to ATP production in each cell line. **(B)** Left, ATP production by OXPHOS and glycolysis

in different ESC and iPSC lines cultured in 2i medium; Right, the relative contribution of OXPHOS and glycolysis to ATP production in each cell line. **(C)** The contribution of OXPHOS to ATP production is increased in ESCs cultured in 2i medium compared with ESCs cultured in traditional serum conditions. **(D)** Left, ATP production by OXPHOS and glycolysis in different MEF cell lines; Right, the relative contribution of OXPHOS and glycolysis to ATP production in each MEF cell line. Results are shown as mean ± SD of 4 replicates from one representative of three independent experiments. *P < 0.05; **P < 0.01; ***P < 0.001; NS, not significant; Student's *t*-test.

Figure S3. Effects of Oligomycin/2-DG on ATP Production and Survival of ESCs (**A**) ATP generation by OXPHOS (OXPHOS-ATP) is inhibited by oligomycin in a dose-dependent manner. Oligomycin inhibits 20% and 50% of maximum OXPHOS-ATP generation (designated as 20%OI and 50%OI) at concentrations of 1 nM and 10 nM, respectively. The OXPHOS-ATP inhibition rates are calculated as follows: $[(OCR_{basal} - OCR_{certain\ oligomycin\ concentration}) / (OCR_{basal} - OCR_{1000\ nM\ oligo})] \times 100\%$. (**B**) ATP generation by glycolysis (glycolysis-ATP) is inhibited by 2-DG in a dose-dependent manner. 2-DG inhibits 20% and 50% of maximum glycolysis-ATP generation (designated as 20%GI and 50%GI) at concentrations of 5 mM and 10 mM, respectively. The glycolysis-ATP inhibition rates are calculated as follows: $[(ECAR_{basal} - ECAR_{certain\ 2\text{-}DG\ concentration}) / (ECAR_{basal} - ECAR_{100\ mM\ 2\text{-}DG})] \times 100\%$. **(C)** 20%OI and 50%GI have similar inhibition effects on total cellular ATP generation. 20%OI, 50%OI, 20%GI and 50%GI accounts for 15.7% ± 5.8%, 36.0% ± 6.2%, 7.7% ± 1.3%, and 15.8% ± 3.6% of total cellular ATP inhibition respectively. Results are shown as mean ± SD from one representative of three independent experiments. 20%OI, 50%OI, n=3; 20%GI, 50%GI, n=4; ns, not significant; Student's *t*-test. (**D**) The total cellular ATP contents were significantly decreased upon inhibition of OXPHOS, inhibition of glycolysis or inhibition of both.

The cellular ATP contents were determined by a Luminescent ATP Detection Kit. Results are shown as mean ± SD of 6 replicates from one representative of three independent experiments, n=6; ***P < 0.001; Student's *t*-test. (**E**) Moderate inhibition of OXPHOS or glycolysis for 48 h does not affect ESC apoptosis. Treatment of ESCs with 2-DG at 100 mM (100%GI) induces ESC death, serving as a positive control. The percentage of surviving cells (Annexin V^-/PI^-) was calculated from flow cytometry data. Results are shown as mean ± SD of 3 replicates from one representative of two independent experiments. ***P < 0.001; Student's *t*-test.

Figure S4. OXPHOS Inhibition Results in More Extensive Gene Expression Reprogram than Glycolysis Inhibition

(A) Volcano plots show the differentially expressed genes (DEGs) between wild-type ESCs and OXPHOS- or glycolysis- inhibited ESCs. (B) OXPHOS inhibition results in more DEGs than glycolysis inhibition. The DEGs shared by both 20%OI and 50%OI ESCs are termed as OXPHOS-related differentially expressed genes (O-DEGs), while the DEGs shared by both 20%GI and 50%GI ESCs are termed as glycolysis-related differentially expressed genes (G-DEGs). (C) Enriched GO term analysis reveals dramatic changes in expression of genes involved in metabolic processes upon OXPHOS inhibition. The gene ratio is indicated by the dot size (the bigger the dot, the greater the ratio) and the significance is indicated by the color of the dot (red, low P value; blue, high P value). 20%OI, 50%OI: inhibition of ATP production by OXPHOS to 20% or 50% of its maximum; 20%GI, 50%GI: inhibition of ATP production by glycolysis to 20% or 50% of its maximum. (**D**) Inhibition of OXPHOS and glycolysis decreases mRNA expression of pluripotency genes. Results are shown as mean ± SD from 3 independent experiments***P < 0.001; Student's t-test.

Figure S5. Oligomycin Directly Inhibits Self-renewal of ESCs and EpiLCs (**A**) OCT4 expression is decreased upon moderate inhibition of OXPHOS but not glycolysis. Immunofluorescence images showing expression of OCT4 (green) in mcherry-labeled ESCs subjected to 20%OI, 50%OI, 20%GI or 50%GI for 48 h. Bars, 50 μm. (**B**) Moderate inhibition of OXPHOS leads to reduced OCT4, SOX2 and NANOG protein expression. Images are representative of 3 independent western blotting experiments. (**C**) Withdrawing oligomycin causes partial recovery of self-renewal in 20%OI and 50%OI ESCs in a time-dependent manner. The ESCs were seeded and subjected to 20%OI or 50%OI treatments for the indicated time, then the oligomycin was withdrawn. After a total culture period of 7 days, alkaline phosphatase staining was used to test the colony formation ability. Left, representative photos of ESC colonies stained by alkaline phosphatase; Right, statistical analysis of the number of alkaline phosphatase-positive colonies. Results are shown as mean ± SD of 3 independent experiments. ***P < 0.001; Student's *t*-test. (**D**) Summary of teratoma formation by ESCs with the indicated treatments in nude mice. (**E**) Inhibition of OXPHOS abolishes the teratoma formation capability of ESCs. Each dot represents the weight of a teratoma formed by ESCs with the indicated treatments. Control, n=30; 20%OI, n=30; 50%OI, n=30; 20%GI, n=22; 50%GI, n=23. (**F**) Images of representative teratomas formed by 20%GI and 50%GI ESCs. Bars, 1 cm. (**G**) Teratomas generated by control, 20%GI and 50%GI ESCs contain three embryonic germ layers. (**H**) Oligomycin inhibits self-renewal of EpiLCs dose-dependently. Left, representative photos of EpiLC colonies stained by alkaline phosphatase; right, statistical analysis of the number of alkaline phosphatase-positive colonies. Results are shown as mean ± SD of 3 replicates from one representative of 3 independent experiments. ***P < 0.001; Student's *t*-test.

Figure S6. Dox-inducible Knockdown of ATP Synthase (**A**) The mRNA expression of ATP5a1 is inhibited by Dox in a dose-dependent manner. Results are shown as mean ± SD from 3 independent experiments. *P < 0.05; ***P < 0.001; Student's *t*-test. (**B**) Inhibition of ATP5a1 expression results in decreased expression of OCT4 (green). Bars, 50 μm.

Figure S7. Genetic Inhibition of Oxidative Phosphorylation Causes Loss of ESC Identity (**A**) Oxygen consumption rate (OCR) measurements on stable ESC lines expressing either scramble shRNA or shRNA targeting ATP5a1 under Dox control. (**B**) The ATP production-related OCR of Tet-shATP5a1 ESC decreases upon Dox treatment. Results are shown as mean ± SD of triplicates from one representative of three independent experiments. **P < 0.01; Student's *t*-test. (**C**) Knockdown of ATP5a1 inhibits ESC self-renewal. Left, representative photos of ESC colonies stained with alkaline phosphatase. Right, statistical analysis of alkaline phosphatase-positive colonies. Results are shown as mean ± SD from 3 independent experiments. ***P < 0.001; Student's *t*-test. (**D**) ATP5a1 knockdown decreases pluripotency gene expression. Results are shown as mean ± SD of 3 independent experiments. *P < 0.05; **P < 0.01; ***P < 0.001; Student's *t*-test. (**E**) Diagram of the chimeric mouse formation assay. (**F**) Knockdown of ATP5a1 decreases the ESC chimera rate. The mCherry-positive cells detected by FACS indicate the number of cells in each chimeric embryo that were derived from the originally injected cells. (**G**) Summary of data from chimeric embryos. Each dot represents the percentage of $mCherry^{+}$ cells in an individual chimeric embryo. Scr, n=36; ATP5a1, n=30. ***P < 0.001; Student's *t*-test.

Figure S8. O-GlcNAcylation of Pluripotency Factors is Essential for ESC Identity (**A**) Heatmap of expression of genes involved in the HBP pathway. (**B**) 50%OI treatment causes decreased O-GlcNAcylation and expression of SOX2 and OCT4 in naïve-state ESCs. (**C**) Statistical analysis of

the western blot results shown in **B**. n=3; *P < 0.05; **P < 0.01; ***P < 0.001; ns, not significant; Student's *t*-test. (**D**) Inhibition of the HBP pathway leads to breakdown of ESC identity. Left, HBP pathway inhibition by Don causes decreased expression of OCT4 and SOX2; right, representative images of ESC colonies stained with alkaline phosphatase. Don, 6-diazo-5-oxo-L-norleucine. (E) Both GlcNAc and glucosamine rescue the decreased O-GlcNAcylation and expression of OCT4 caused by OXPHOS inhibition. (**F**) Compromised expression of pluripotency genes led by OXPHOS inhibition is partially rescued by GlcNAc. Values displayed correspond to the expression level in the indicated sample scaled by the mean expression of each gene across samples. (**G**) OXPHOS inhibition causes altered expression of genes in the whole protein-coding transcriptome, and this effect is partially rescued by GlcNAc. Values displayed correspond to the expression level in the indicated sample scaled by the mean expression of each gene across samples. (**H**) Inhibition of OXPHOS rather than glycolysis reduces O-GlcNAcylation and expression of SOX2 in the inner cell mass of *ex vivo*-cultured blastocysts. These effects are ameliorated by adding GlcNAc. Confocal immunofluorescence microscopy was carried out using antibodies specific to O-GlcNAc (green) and SOX2 (red). The nuclei were stained with DAPI. Bars, 25 μm. (**I**) Statistical analysis of the immunofluorescence microscopy results shown in **H**. Control, n=10; 20%OI, n=11; 20%OI+GlcNAC, n=10; 20%GI, n=7; 50%OI, n=9; 50%OI+GlcNAC, n=9; 50%GI, n=10; *P < 0.05; **P < 0.01; ***P < 0.001; ns, not significant; Student's *t*-test. (**J**) The proliferation capacity of OXPHOS inhibited ESCs can not be rescued by adding GlcNAc. Results are shown as mean ± SD of 3 replicates from one representative of three independent experiments. ***P < 0.001; ns, not significant; Student's *t*-test. (**K**) The deficient chimera contribution of OXPHOS-inhibited ESCs can be rescued by adding GlcNAc. Above, the GFP-positive cells detected by FACS indicate the

number of cells in each chimeric embryo that were derived from the originally injected cells. Below, summary of data from chimeric embryos. Each dot represents the percentage of GFP^+ cells in an individual chimeric embryo. control, n=8; 20%OI, n=9; 20%OI+GlcNAc, n=8. $^{**}P < 0.01$; Student's *t*-test.

Figure S9. OXPHOS Inhibition Induces ESCs into a State Which is Different to the Diapause- or Primed- State (**A**) Diffusion map of ESCs, 20%OI- or 50%OI- treated ESCs and ESCs (2i/LIF), EPIs(E4.5), diapause EPIs or EPIs(E5.5) shows that OXPHOS inhibition induces ESCs into a unique state, which is closer to the diapause- state than to the primed- state (EPIs, epiblasts). (**B**) Scatterplots of gene expression levels that are significantly changed in the comparison of 20%OI/WT and Diapause/E4.5 (top left), 20%OI/WT and Primed(E5.5)/E4.5 (top right), 50%OI/WT and Diapause/E4.5 (bottom left) or 50%OI/WT and Primed(E5.5)/E4.5 (bottom right). The x axis shows the $\log_2$ fold change ($\log_2$ Fc) between 20%OI-(or 50%OI-) ESCs and control ESCs; the y axis shows the $\log_2$ Fc between diapause epiblasts (or E5.5 primed epiblasts) and E4.5 epiblasts. The dots represent the set of all genes. Differentially expressed genes ($|FC|\geq 1.5$ and $p\leq 0.05$) are shown as red dots. The numbers of DEGs (red) and total genes (gray) are labeled in every corresponding quadrant. The linear equation is fitted by all the dots in the four quadrants, and the slope of this linear fitting equation partially reflects the correlation of the x and y axes. R^2 values display correlation coefficients. Data for ESCs(2i/LIF), EPIs(E4.5), diapause EPIs and EPIs(E5.5) are from Boroviak et al. 2015.

Figure S10. Network Integration of Metabolome and Transcriptome Data Reveals that Inhibition of OXPHOS Leads to Defective UDP-GlcNAc Biosynthesis A global combined network that connects metabolite nodes to reaction nodes was constructed based on the detected

metabolites and the corresponding enzymes. The node of each reaction is connected to its respective substrates and products (solid circles). Enzymes that catalyze representative reactions are shown (diamonds). The relative levels of metabolites and enzymes in 20%OI ESCs compared to control ESCs is indicated by different colors (red, upregulated; green, downregulated; blue, not measured), and the significance is indicated by dot size (the bigger the more significant).

Figure S11. UDP-GlcNAc Links Oxidative Phosphorylation to Pluripotency ESC mitochondria have a high ATP generation capacity. Mitochondrial respiration produces the majority of cellular ATP in ESC, and couples with the hexosamine biosynthesis pathway to generate UDP-GlcNAc for O-GlcNAcylation of pluripotency factors like OCT4 and SOX2, thereby safeguarding ESC identity. Moderate inhibition of mitochondrial respiration leads to incomplete catabolism of glucose together with abnormal metabolism of glutamine, nucleotides, and acetyl-CoA, resulting in decreased UDP-GlcNAc generation and compromised pluripotency. This effect can be ameliorated by direct supplementation of GlcNAc.

Figure S1. Determination of the Contribution of Oxidative Phosphorylation and Glycolysis to ATP Generation in Mouse ESC, EpiLC, NSC, MEF and HL-1

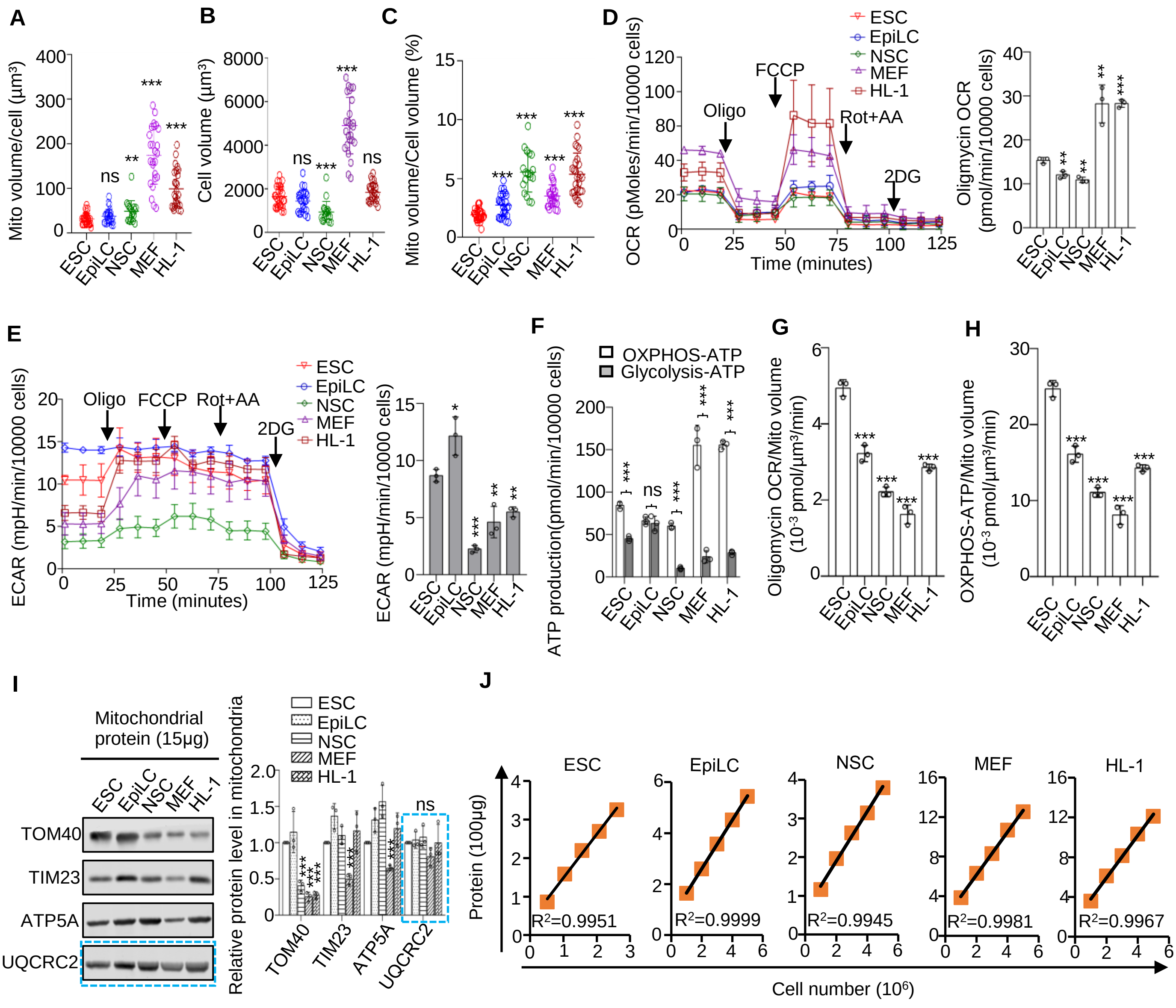

K

Cell line	Protein(pg) /cell
ESC	257.43±18.28
EpiLC	222.05±3.80
NSC	146.14±1.19
MEF	535.00±0.56
HL-1	563.33±2.22

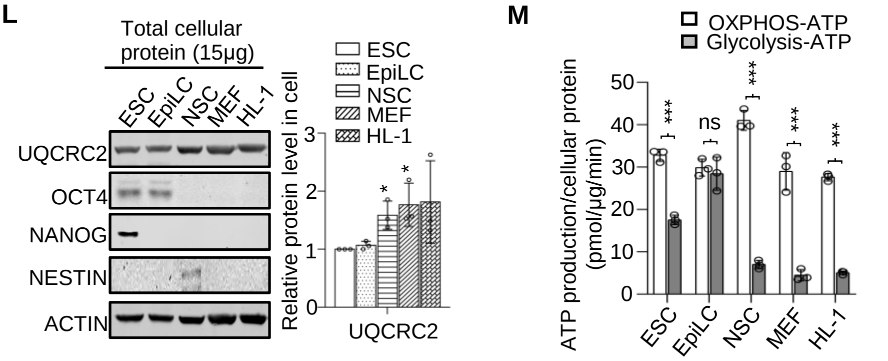

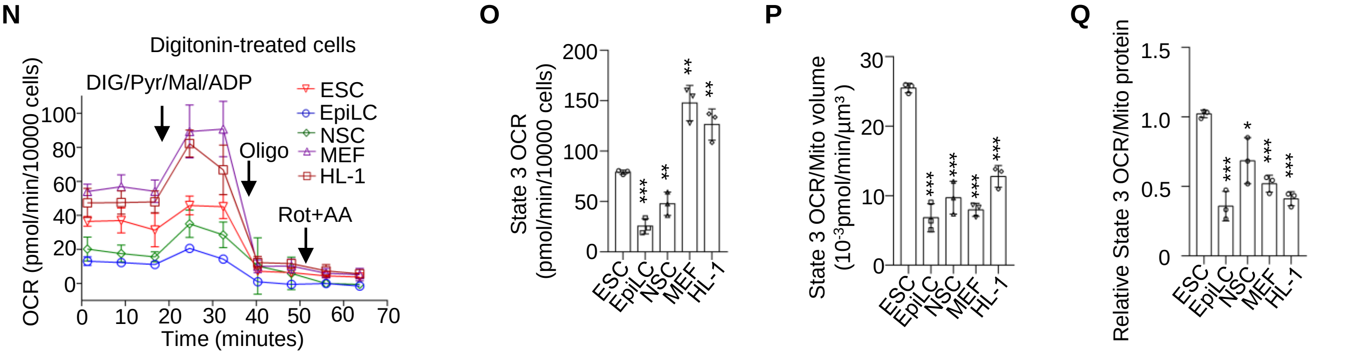

Figure S1. Determination of the Contribution of Oxidative Phosphorylation and Glycolysis to ATP Generation in Mouse ESCs, EpiLCs, NSCs, MEFs and HL-1 cells

(**A**) The total mitochondrial volume in an ESC (31.05 ± 12.45 μm^3) is significantly smaller than that in an NSC (49.02 ± 23.46 μm^3), an MEF (174 ± 64.68 μm^3) or an HL-1 cell (99.03 ± 42.5 μm^3), and is similar to that in an EpiLC (37.4 ± 16.08 μm^3). The total mitochondrial volume in individual ESCs, EpiLCs, NSCs, MEFs and HL-1 cells was determined by SIM. Results are shown as mean ± SD; ESC, n=29; EpiLC, n=25; NSC, n=19; MEF, n=25; HL-1, n=26; **P < 0.01; ***P < 0.001; ns, not significant; Student's *t*-test. (**B**) An ESC (1633 ± 476.7 μm^3), which is similar in volume to an EpiLC (1435 ± 491.7 μm^3) and an HL-1 cell (1830 ± 416.8 μm^3), is smaller than an MEF (4895 ± 1296 μm^3) but is larger than an NSC (932.9 ± 476.3 μm^3). The volumes of individual ESCs, EpiLCs, NSCs, MEFs and HL-1 cells were determined by SIM. Data are shown as mean ± SD; ESC, n=29; EpiLC, n=25; NSC, n=19; MEF, n=25; HL-1, n=26; ***P < 0.001; ns, not significant; Student's *t*-test. (**C**) The ratio of total mitochondrial volume to cell volume in ESCs (1.9% ± 0.5%) is smaller than in EpiLCs (2.72% ± 0.98%), NSCs (5.5% ± 1.68%), MEFs (3.5% ± 0.94%) and HL-1 cells (5.36% ± 1.79%). Results are shown as mean ± SD; ESC, n=29; EpiLC, n=25; NSC, n=19; MEF, n=25; HL-1, n=26; ***P < 0.001; Student's *t*-test. (**D**) Left, Oxygen consumption rate (OCR) measured by Seahorse Extracellular Flux Assay; right, oligomycin-sensitive oxygen consumption rates (Oligomycin OCR) in naïve ESCs, EpiLCs, NSCs, MEFs and HL-1 cells. Results are shown as mean ± SD of three independent experiments; **P < 0.01; ***P < 0.001; Student's *t*-test. (**E**) Left, Extracellular acidification rate (ECAR) measured by Seahorse Extracellular Flux Assay; right, glycolytic rate (ECAR) in naïve ESCs, EpiLCs, NSCs, MEFs and HL-1 cells. Results are shown as mean ± SD of three independent experiments; *P < 0.05; **P < 0.01; ***P < 0.001; Student's *t*-test. (**F**) ATP production by oxidative phosphorylation (OXPHOS-ATP) or glycolysis (Glycolysis-ATP) in 10000 naïve ESCs, EpiLCs, NSCs, MEFs and HL-1 cells. The oligomycin-sensitive oxygen consumption rate is converted into the OXPHOS ATP production rate using a P/O ratio of 2.75, and ATP production by glycolysis is determined by a one-to-one relationship between the generation of protons and ATP. The ratio of proton production rate to ECAR (PPR/ECAR) was determined as 5.198 using the Seahorse XF24 analyzer based on the buffer capacity of the assay medium. Results are shown as mean ± SD of three independent experiments. n=3; ***P < 0.001; ns, not significant; Student's *t*-test. (**G**) The rate of oligomycin-sensitive oxygen consumption (Oligomycin OCR/Mito volume) is significantly higher in ESCs than in EpiLCs, NSCs, MEFs and HL-1 cells. Values are normalized to mitochondrial volume. Results are shown as mean ± SD of one representative from three independent experiments. n=3; ***P < 0.001; Student's *t*-test. (**H**) The OXPHOS-ATP generation (OXPHOS-ATP/Mito volume) is significantly higher in ESCs than in EpiLCs, NSCs, MEFs or HL-1 cells. Values are normalized to mitochondrial volume. Results are shown as mean ± SD of one representative from three independent experiments. n=3; ***P < 0.001; Student's *t*-test.(**I**) The level of UQCRC2 protein is constant in mitochondria from naïve ESCs, EpiLCs, NSCs, MEFs and HL-1 cells. The data are normalized to equal mitochondrial protein mass. Left, the level of TOM40, TIM23, ATP5A and UQCRC2 were detected by western blotting in 15 µg mitochondrial proteins isolated from naïve ESCs, EpiLCs, NSCs, MEFs and HL-1 cells. Right, results are shown as mean ± SD of three independent experiments. n=3; ***P < 0.001; ns, not significant; Student's *t*-test. (**J**) Titration of cellular protein quantity in naïve ESCs, EpiLCs, NSCs, MEFs and HL-1 cells. (**K**) Absolute protein quantity in an individual naïve ESC, EpiLC, NSC, MEF and HL-1 cell. (**L**) The relative levels of UQCRC2 in an equal mass of total cellular protein. Left, the expression levels of UQCRC2, OCT4, NANOG, NESTIN and ACTIN in 15 µg cellular proteins from naïve ESCs, EpiLCs, NSCs, MEFs and HL-1 cardiomyocytes were detected by western blotting; right, results are shown as mean ± SD of three independent experiments. n=3; *P < 0.05; Student's *t*-test. (**M**) ATP production by OXPHOS or glycolysis in naïve ESCs, EpiLCs, NSCs, MEFs and HL-1 cells. Data are normalized to an equal mass of cellular protein. Results are shown as mean ± SD of three independent experiments. n=3; ***P < 0.001; ns, not significant; Student's *t*-test. (**N**) OCR was measured by Seahorse Extracellular Flux Assay using digitonin-permeabilized cells fed complex-specific substrates of the preceding complex in the electron transport chain. DIG, digitonin; Pyr, pyruvate; Mal, malic acid; ADP, Adenosine-5'-diphosphate; Oligo, oligomycin; Rot, rotenone. (**O**) State 3 OCR (OCR values measured after injection of DIG/Pyr/Mal/ADP) in naïve ESCs is significantly higher than in EpiLCs and NSCs, and lower than in MEFs and HL-1 cells, when the data are normalized to equal numbers of cells. Results are shown as mean ± SD of three independent experiments. n=3; **P < 0.01; ***P < 0.001; Student's *t*-test. (**P**) State 3 OCR in naïve ESC is significantly higher than that in EpiLCs, NSCs, MEFs or HL-1 cells when the data are normalized to equal mitochondrial volume. Results are shown as mean ± SD of three independent experiments. n=3; *P < 0.05; **P < 0.01; Student's *t*-test. (**Q**) Relative state 3 OCR in naïve ESCs is significantly higher than in EpiLCs, NSCs, MEFs and HL-1 cells when the data are normalized to equal mitochondrial protein mass. Results are shown as mean ± SD of three independent experiments. n=3; *P < 0.05; ***P < 0.001; Student's *t*-test.

Figure S2. ATP generation by OXPHOS and Glycolysis in Different Somatic Cell and Pluripotent Stem Cell Lines

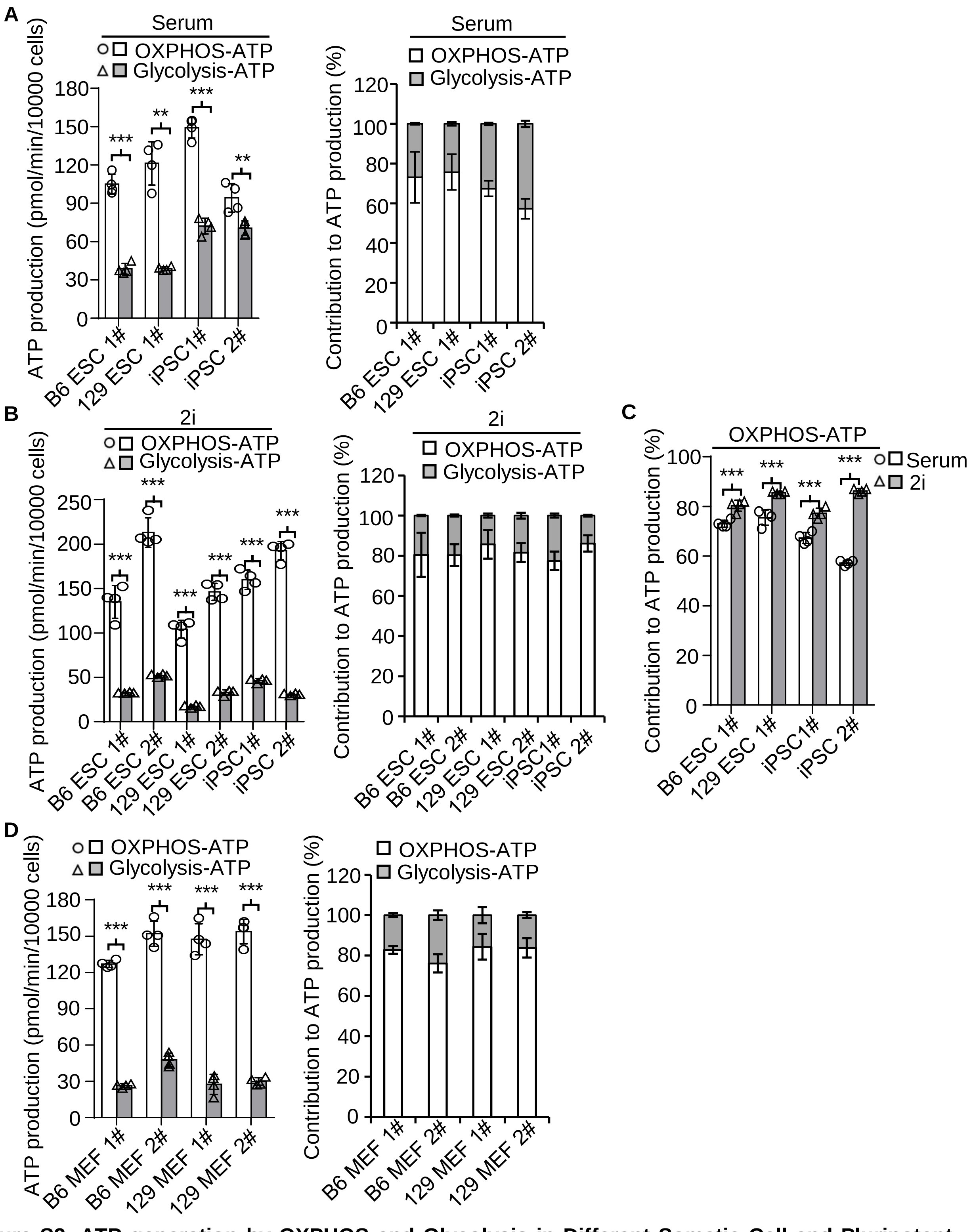

Figure S2. ATP generation by OXPHOS and Glycolysis in Different Somatic Cell and Pluripotent Stem Cell Lines

(A) Left, ATP production by OXPHOS and glycolysis in different ESC and iPSC lines cultured in ESC medium suppled with fetal bovine serum; Right, the relative contribution of OXPHOS and glycolysis to ATP production in each cell line. **(B)** Left, ATP production by OXPHOS and glycolysis in different ESC and iPSC lines cultured in 2i medium; Right, the relative contribution of OXPHOS and glycolysis to ATP production in each cell line. **(C)** The contribution of OXPHOS to ATP production is increased in ESCs cultured in 2i medium compared with ESCs cultured in traditional serum conditions. **(D)** Left, ATP production by OXPHOS and glycolysis in different MEF cell lines; Right, the relative contribution of OXPHOS and glycolysis to ATP production in each MEF cell line. Results are shown as mean ± SD of 4 replicates from one representative of three independent experiments. *P < 0.05; **P < 0.01; ***P < 0.001; NS, not significant; Student's *t*-test.

Figure S3. Effects of Oligomycin/2-DG on ATP Production and Survival of ESCs

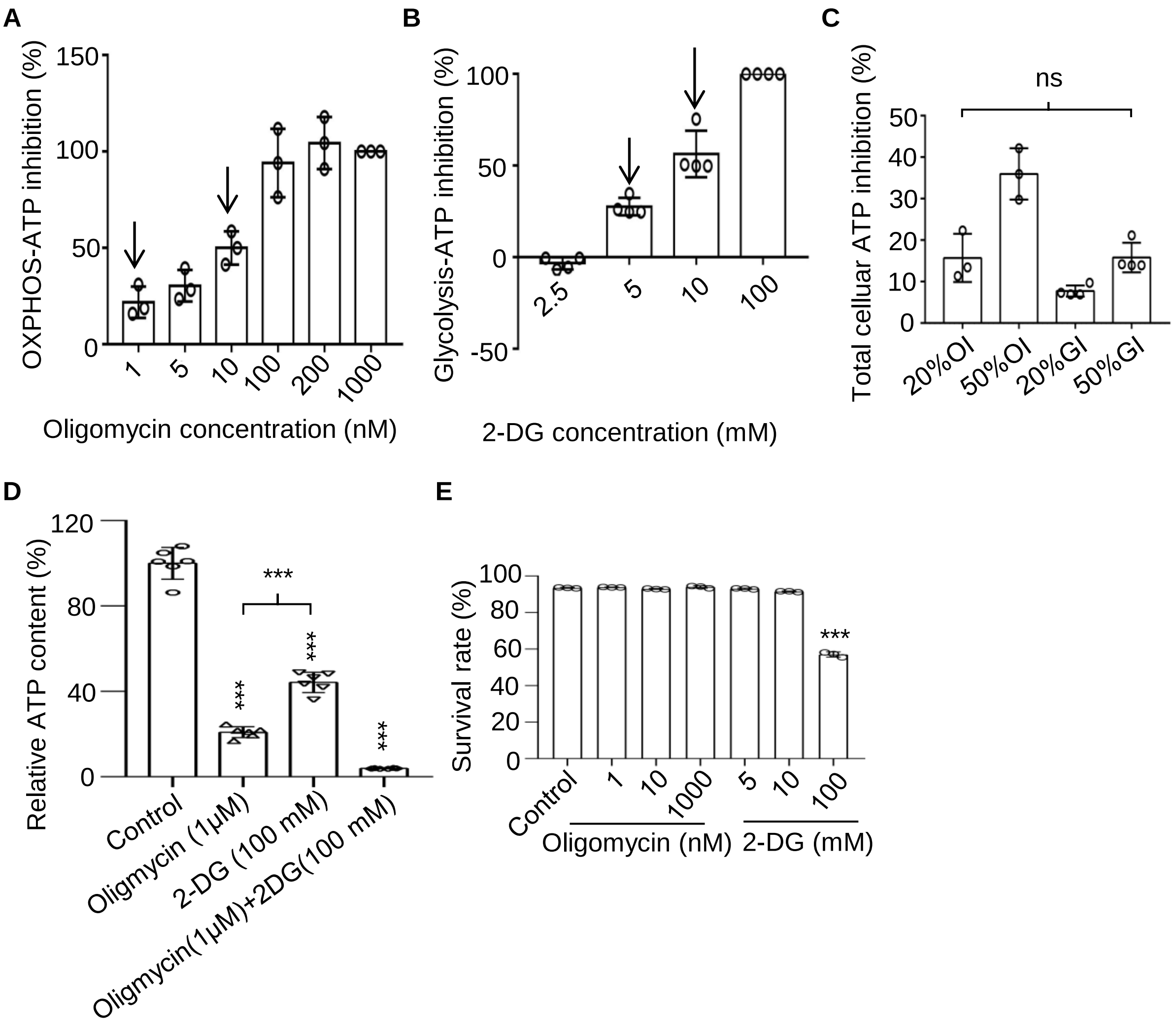

Figure S3. Effects of Oligomycin/2-DG on ATP Production and Survival of ESCs (**A**) ATP generation by OXPHOS (OXPHOS-ATP) is inhibited by oligomycin in a dose-dependent manner. Oligomycin inhibits 20% and 50% of maximum OXPHOS-ATP generation (designated as 20%OI and 50%OI) at concentrations of 1 nM and 10 nM, respectively. The OXPHOS-ATP inhibition rates are calculated as follows: $[(OCR_{basal} - OCR_{certain\ oligomycin\ concentration}) / (OCR_{basal} - OCR_{1000\ nM\ oligo})] \times 100\%$. (**B**) ATP generation by glycolysis (glycolysis-ATP) is inhibited by 2-DG in a dose-dependent manner. 2-DG inhibits 20% and 50% of maximum glycolysis-ATP generation (designated as 20%GI and 50%GI) at concentrations of 5 mM and 10 mM, respectively. The glycolysis-ATP inhibition rates are calculated as follows: $[(ECAR_{basal} - ECAR_{certain\ 2\text{-}DG\ concentration}) / (ECAR_{basal} - ECAR_{100\ mM\ 2\text{-}DG})] \times 100\%$. **(C)** 20%OI and 50%GI have similar inhibition effects on total cellular ATP generation. 20%OI, 50%OI, 20%GI and 50%GI accounts for 15.7% ± 5.8%, 36.0% ± 6.2%, 7.7% ± 1.3%, and 15.8% ± 3.6% of total cellular ATP inhibition respectively. Results are shown as mean ± SD from one representative of three independent experiments. 20%OI, 50%OI, n=3; 20%GI, 50%GI, n=4; ns, not significant; Student's *t*-test. (**D**) The total cellular ATP contents were significantly decreased upon inhibition of OXPHOS, inhibition of glycolysis or inhibition of both. The cellular ATP contents were determined by a Luminescent ATP Detection Kit. Results are shown as mean ± SD of 6 replicates from one representative of three independent experiments, n=6; ***P < 0.001; Student's *t*-test. (**E**) Moderate inhibition of OXPHOS or glycolysis for 48 h does not affect ESC apoptosis. Treatment of ESCs with 2-DG at 100 mM (100%GI) induces ESC death, serving as a positive control. The percentage of surviving cells (Annexin V^-/PI^-) was calculated from flow cytometry data. Results are shown as mean ± SD of 3 replicates from one representative of two independent experiments. ***P < 0.001; Student's *t*-test.

Figure S4. OXPHOS Inhibition Results in More Extensive Gene Expression Reprogram than Glycolysis Inhibition

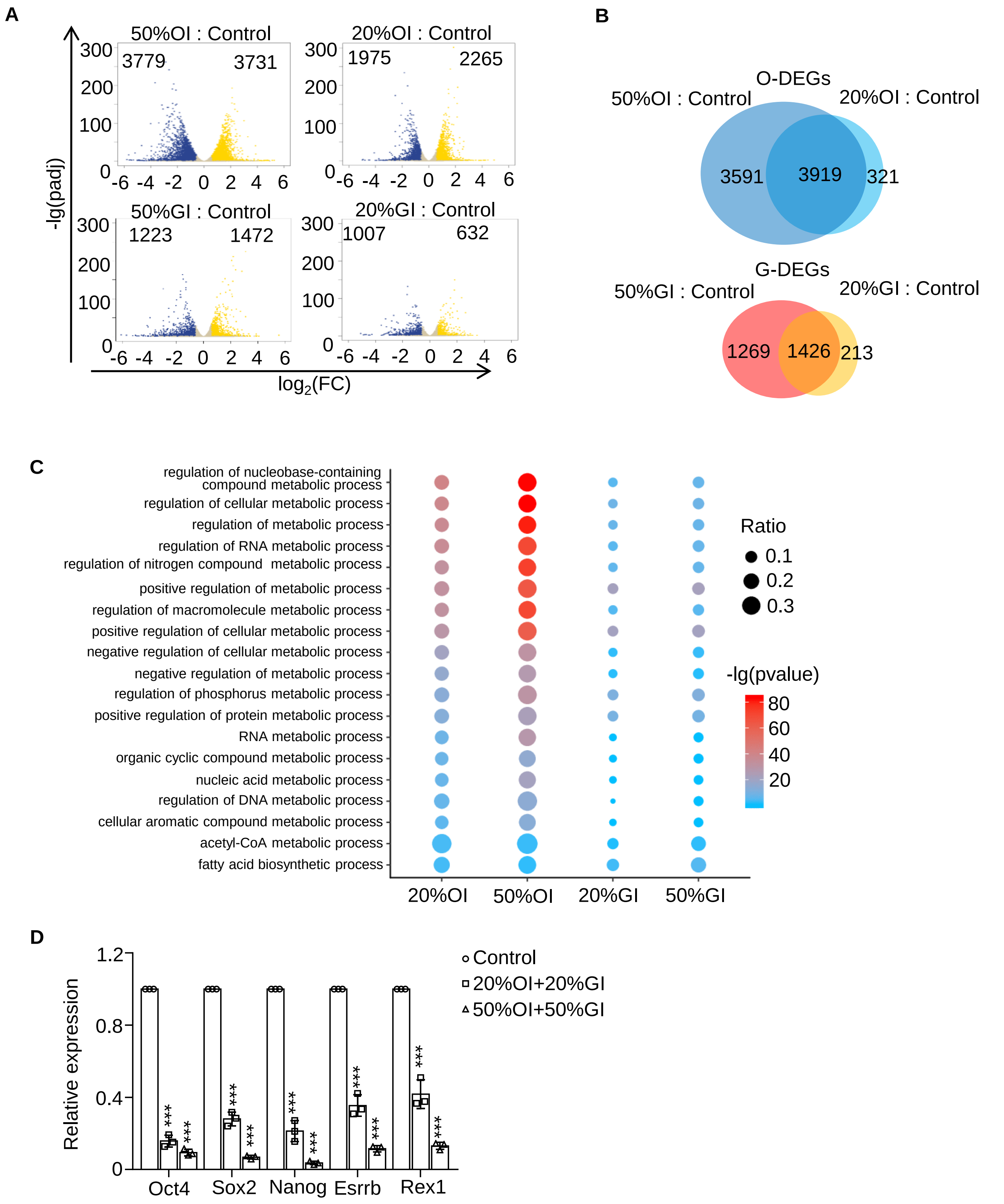

Fig S4. OXPHOS Inhibition Results in More Extensive Gene Expression Reprogram than Glycolysis Inhibition (**A**) Volcano plots show the differentially expressed genes (DEGs) between wild-type ESCs and OXPHOS- or glycolysis- inhibited ESCs. (**B**) OXPHOS inhibition results in more DEGs than glycolysis inhibition. The DEGs shared by both 20%OI and 50%OI ESCs are termed as OXPHOS-related differentially expressed genes (O-DEGs), while the DEGs shared by both 20%GI and 50%GI ESCs are termed as glycolysis-related differentially expressed genes (G-DEGs). (**C**) Enriched GO term analysis reveals dramatic changes in expression of genes involved in metabolic processes upon OXPHOS inhibition. The gene ratio is indicated by the dot size (the bigger the dot, the greater the ratio) and the significance is indicated by the color of the dot (red, low P value; blue, high P value). 20%OI, 50%OI: inhibition of ATP production by OXPHOS to 20% or 50% of its maximum; 20%GI, 50%GI: inhibition of ATP production by glycolysis to 20% or 50% of its maximum. (**D**) Inhibition of OXPHOS and glycolysis decreases mRNA expression of pluripotency genes. Results are shown as mean ± SD from 3 independent experiments∗∗∗P < 0.001; Student’s t-test.

Figure S5. Oligomycin Directly Inhibits Self-renewal of ESC and EpiLC

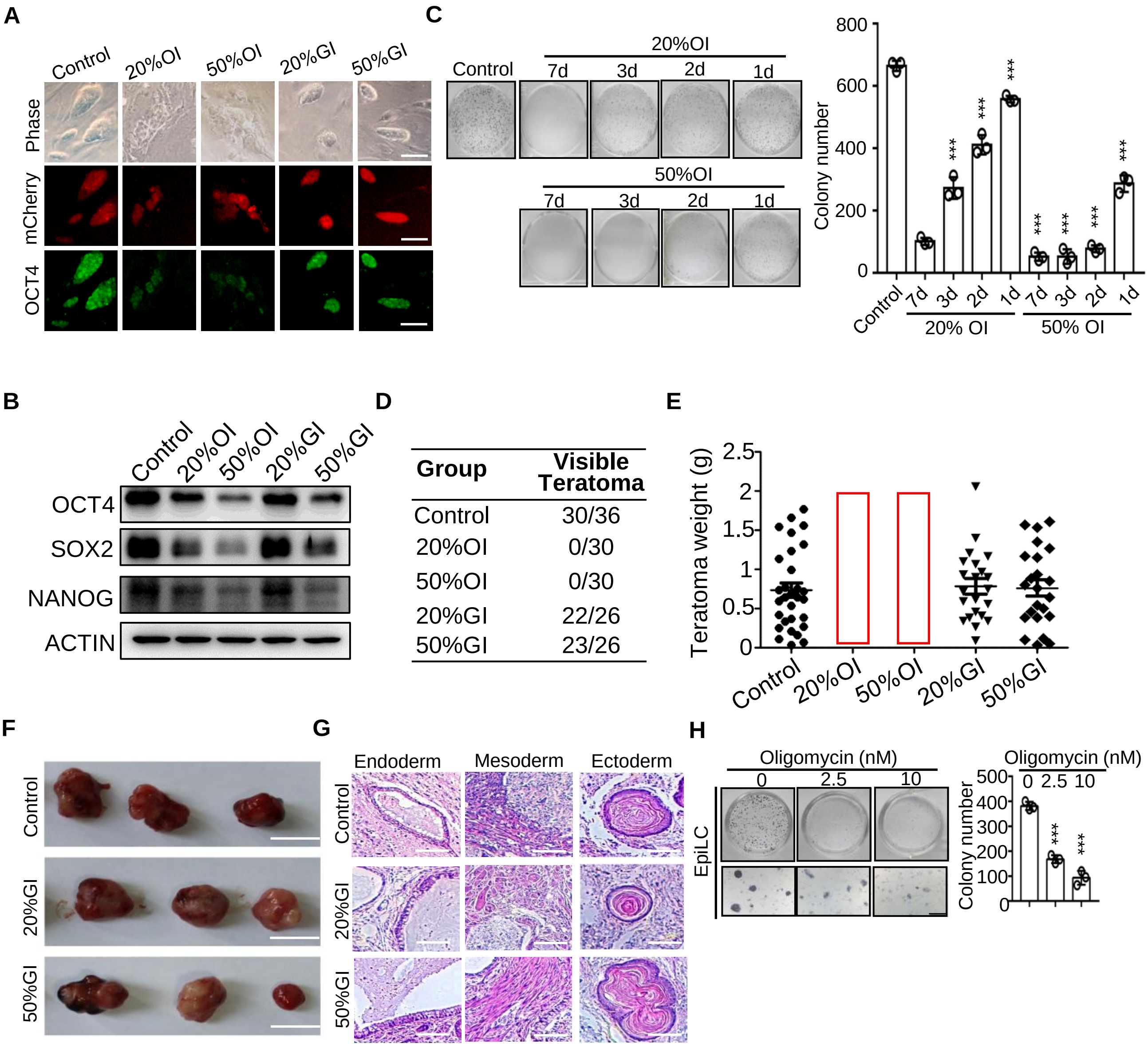

Figure S5. Oligomycin Directly Inhibits Self-renewal of ESCs and EpiLCs (**A**) OCT4 expression is decreased upon moderate inhibition of OXPHOS but not glycolysis. Immunofluorescence images showing expression of OCT4 (green) in mcherry-labeled ESCs subjected to 20%OI, 50%OI, 20%GI or 50%GI for 48 h. Bars, 50 µm. (**B**) Moderate inhibition of OXPHOS leads to reduced OCT4, SOX2 and NANOG protein expression. Images are representative of 3 independent western blotting experiments. (**C**) Withdrawing oligomycin causes partial recovery of self-renewal in 20%OI and 50%OI ESCs in a time-dependent manner. The ESCs were seeded and subjected to 20%OI or 50%OI treatments for the indicated time, then the oligomycin was withdrawn. After a total culture period of 7 days, alkaline phosphatase staining was used to test the colony formation ability. Left, representative photos of ESC colonies stained by alkaline phosphatase; Right, statistical analysis of the number of alkaline phosphatase-positive colonies. Results are shown as mean ± SD of 3 independent experiments. ***P < 0.001; Student's *t*-test. (**D**) Summary of teratoma formation by ESCs with the indicated treatments in nude mice. (**E**) Inhibition of OXPHOS abolishes the teratoma formation capability of ESCs. Each dot represents the weight of a teratoma formed by ESCs with the indicated treatments. Control, n=30; 20%OI, n=30; 50%OI, n=30; 20%GI, n=22; 50%GI, n=23. (**F**) Images of representative teratomas formed by 20%GI and 50%GI ESCs. Bars, 1 cm. (**G**) Teratomas generated by control, 20%GI and 50%GI ESCs contain three embryonic germ layers. (**H**) Oligomycin inhibits self-renewal of EpiLCs dose-dependently. Left, representative photos of EpiLC colonies stained by alkaline phosphatase; right, statistical analysis of the number of alkaline phosphatase-positive colonies. Results are shown as mean ± SD of 3 replicates from one representative of 3 independent experiments. ***P < 0.001; Student's *t*-test.

Figure S6. Dox-inducible Knockdown of ATP Synthase

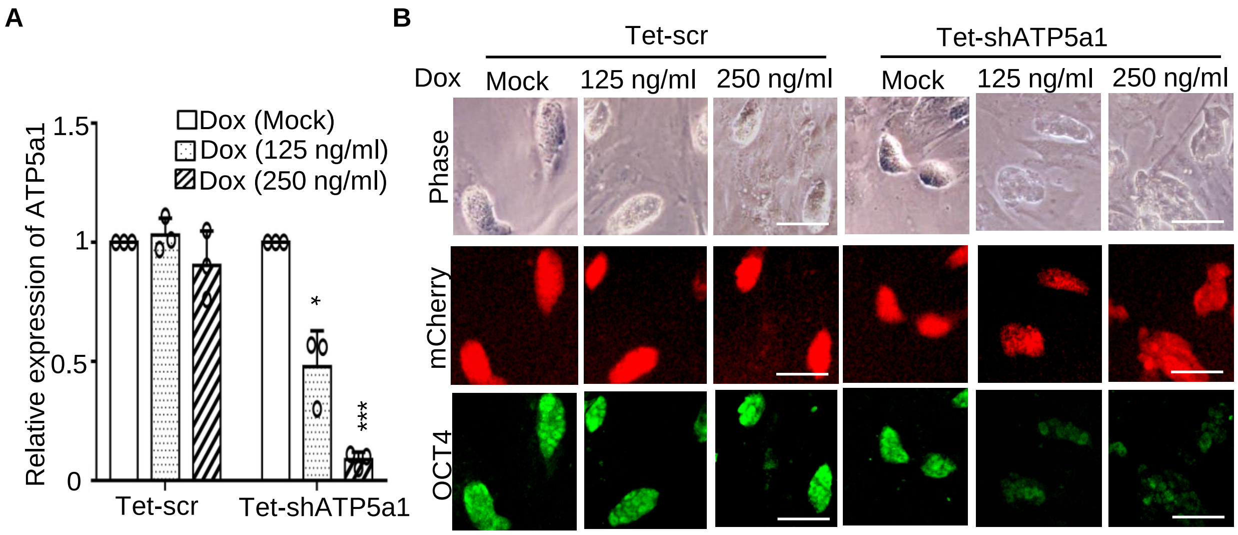

Figure S6. Dox-inducible Knockdown of ATP Synthase (**A**) The mRNA expression of ATP5a1 is inhibited by Dox in a dose-dependent manner. Results are shown as mean ± SD from 3 independent experiments. *P < 0.05; ***P < 0.001; Student's *t*-test. (**B**) Inhibition of ATP5a1 expression results in decreased expression of OCT4 (green). Bars, 50 μm.

Figure S7. Genetic Inhibition of Oxidative Phosphorylation Causes Loss of ESC Identity

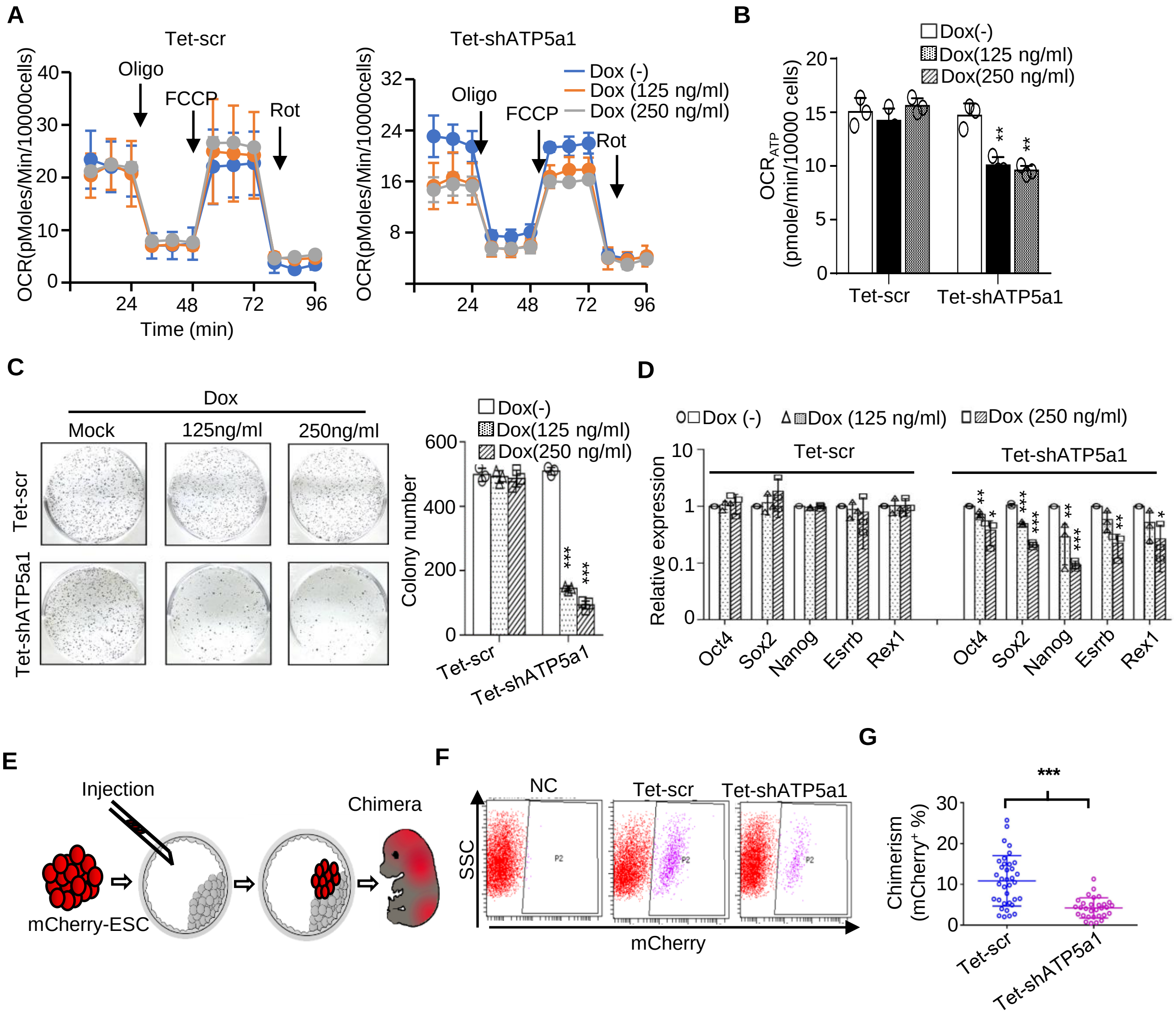

Figure S7. Genetic Inhibition of Oxidative Phosphorylation Causes Loss of ESC Identity (**A**) Oxygen consumption rate (OCR) measurements on stable ESC lines expressing either scramble shRNA or shRNA targeting ATP5a1 under Dox control. (**B**) The ATP production-related OCR of Tet-shATP5a1 ESC decreases upon Dox treatment. Results are shown as mean ± SD of triplicates from one representative of three independent experiments. **P < 0.01; Student's *t*-test. (**C**) Knockdown of ATP5a1 inhibits ESC self-renewal. Left, representative photos of ESC colonies stained with alkaline phosphatase. Right, statistical analysis of alkaline phosphatase-positive colonies. Results are shown as mean ± SD from 3 independent experiments. ***P < 0.001; Student's *t*-test. (**D**) ATP5a1 knockdown decreases pluripotency gene expression. Results are shown as mean ± SD of 3 independent experiments. *P < 0.05; **P < 0.01; ***P < 0.001; Student's *t*-test. (**E**) Diagram of the chimeric mouse formation assay. (**F**) Knockdown of ATP5a1 decreases the ESC chimera rate. The mCherry-positive cells detected by FACS indicate the number of cells in each chimeric embryo that were derived from the originally injected cells. (**G**) Summary of data from chimeric embryos. Each dot represents the percentage of mCherry^{+} cells in an individual chimeric embryo. Scr, n=36; ATP5a1, n=30. ***P < 0.001; Student's *t*-test.

Figure S8. O-GlcNAcylation of Pluripotency Factors is Essential for ESC Identity

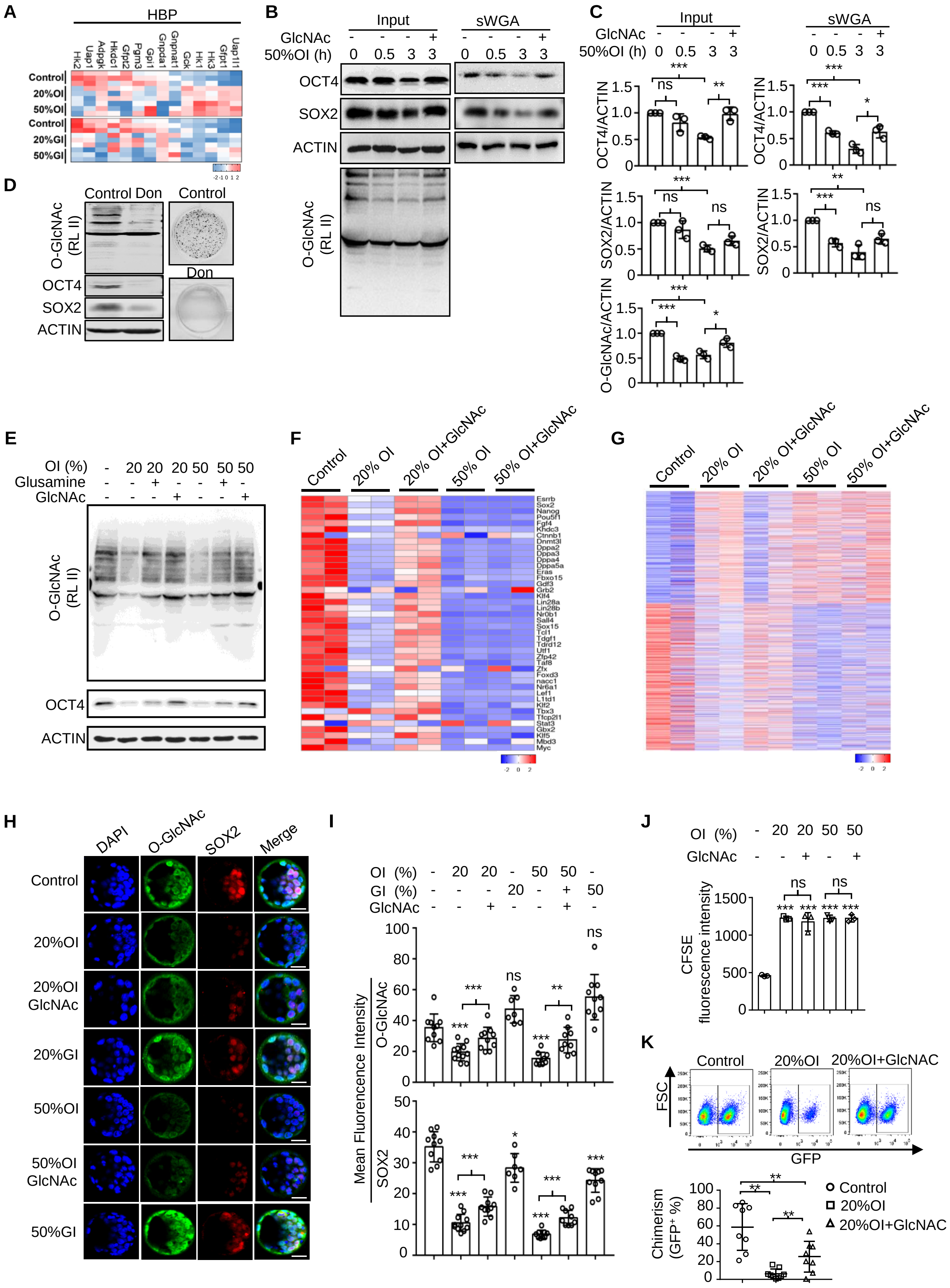

Figure S8. O-GlcNAcylation of Pluripotency Factors is Essential for ESC Identity (**A**) Heatmap of expression of genes involved in the HBP pathway. (**B**) 50%OI treatment causes decreased O-GlcNAcylation and expression of SOX2 and OCT4 in naïve-state ESCs. (**C**) Statistical analysis of the western blot results shown in **B**. n=3; *P < 0.05; **P < 0.01; ***P < 0.001; ns, not significant; Student's *t*-test. (**D**) Inhibition of the HBP pathway leads to breakdown of ESC identity. Left, HBP pathway inhibition by Don causes decreased expression of OCT4 and SOX2; right, representative images of ESC colonies stained with alkaline phosphatase. Don, 6-diazo-5-oxo-L-norleucine. (E) Both GlcNAc and glucosamine rescue the decreased O-GlcNAcylation and expression of OCT4 caused by OXPHOS inhibition. (**F**) Compromised expression of pluripotency genes led by OXPHOS inhibition is partially rescued by GlcNAc. Values displayed correspond to the expression level in the indicated sample scaled by the mean expression of each gene across samples. (**G**) OXPHOS inhibition causes altered expression of genes in the whole protein-coding transcriptome, and this effect is partially rescued by GlcNAc. Values displayed correspond to the expression level in the indicated sample scaled by the mean expression of each gene across samples. (**H**) Inhibition of OXPHOS rather than glycolysis reduces O-GlcNAcylation and expression of SOX2 in the inner cell mass of *ex vivo*-cultured blastocysts. These effects are ameliorated by adding GlcNAc. Confocal immunofluorescence microscopy was carried out using antibodies specific to O-GlcNAc (green) and SOX2 (red). The nuclei were stained with DAPI. Bars, 25 µm. (**I**) Statistical analysis of the immunofluorescence microscopy results shown in **H**. Control, n=10; 20%OI, n=11; 20%OI+GlcNAC, n=10; 20%GI, n=7; 50%OI, n=9; 50%OI+GlcNAC, n=9; 50%GI, n=10; *P < 0.05; **P < 0.01; ***P < 0.001; ns, not significant; Student's *t*-test. (J) The proliferation capacity of OXPHOS inhibited ESCs can not be rescued by adding GlcNAc. Results are shown as mean ± SD of 3 replicates from one representative of three independent experiments. ***P < 0.001; ns, not significant; Student's *t*-test. (K) The deficient chimera contribution of OXPHOS-inhibited ESCs can be rescued by adding GlcNAc. Above, the GFP-positive cells detected by FACS indicate the number of cells in each chimeric embryo that were derived from the originally injected cells. Below, summary of data from chimeric embryos. Each dot represents the percentage of GFP^+ cells in an individual chimeric embryo. control, n=8; 20%OI, n=9; 20%OI+ GlcNAc, n=8. **P < 0.01; Student's *t*-test.

Figure S9. OXPHOS Inhibition Induces ESCs to A State Which is Different to Diapause- or Primed- State

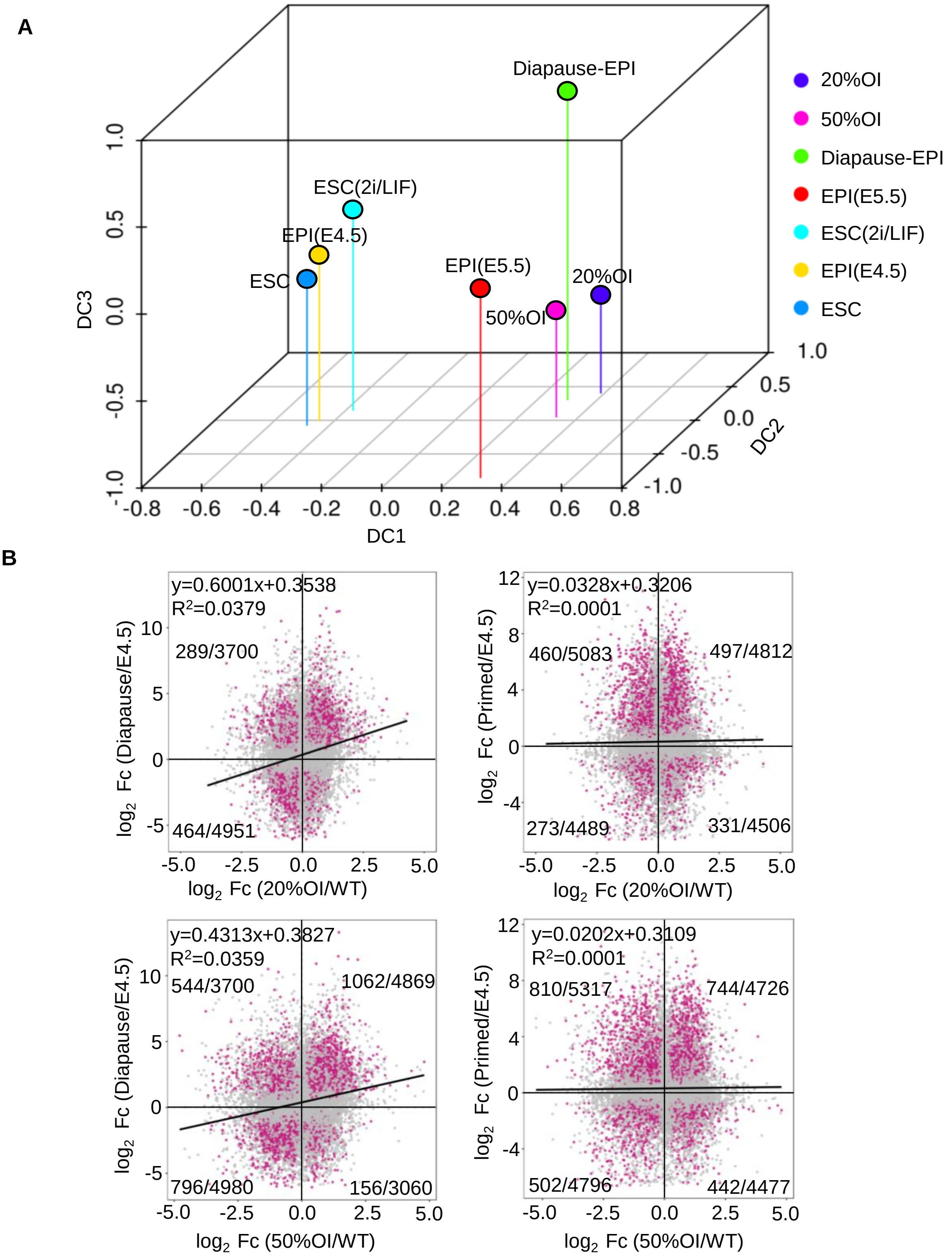

Figure S9. OXPHOS Inhibition Induces ESCs into a State Which is Different to the Diapause- or Primed- State (**A**) Diffusion map of ESCs, 20%OI- or 50%OI- treated ESCs and ESCs(2i/LIF), EPIs(E4.5), diapause EPIs or EPIs(E5.5) shows that OXPHOS inhibition induces ESCs into a unique state, which is closer to the diapause- state than to the primed- state (EPIs, epiblasts). (**B**) Scatterplots of gene expression levels that are significantly changed in the comparison of 20%OI/WT and Diapause/E4.5 (top left), 20%OI/WT and Primed(E5.5)/E4.5 (top right), 50%OI/WT and Diapause/E4.5 (bottom left) or 50%OI/WT and Primed(E5.5)/E4.5 (bottom right). The x axis shows the $\log_2$ fold change ($\log_2$ Fc) between 20%OI-(or 50%OI-) ESCs and control ESCs; the y axis shows the $\log_2$ Fc between diapause epiblasts (or E5.5 primed epiblasts) and E4.5 epiblasts. The dots represent the set of all genes. Differentially expressed genes ($|FC|\geq1.5$ and $p\leq0.05$) are shown as red dots. The numbers of DEGs (red) and total genes (gray) are labeled in every corresponding quadrant. The linear equation is fitted by all the dots in the four quadrants, and the slope of this linear fitting equation partially reflects the correlation of the x and y axes. R^2 values display correlation coefficients. Data for ESCs(2i/LIF), EPIs(E4.5), diapause EPIs and EPIs(E5.5) are from Boroviak et al. 2015.

Figure S10. Network Integration of Metabolome and Transcriptome Data Reveals that Inhibition of OXPHOS Leads to Defective UDP-GlcNAc Biosynthesis

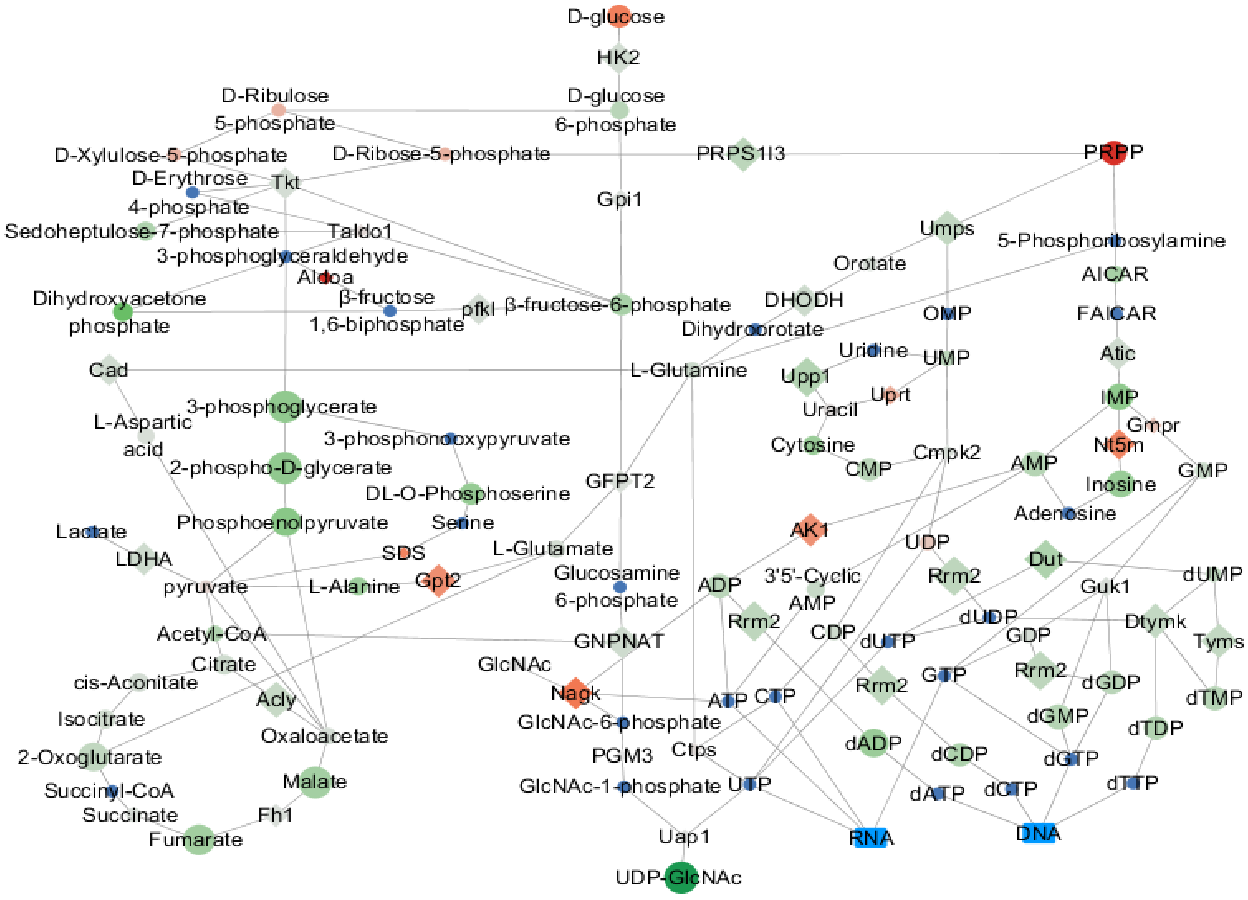

Figure S10. Network Integration of Metabolome and Transcriptome Data Reveals that Inhibition of OXPHOS Leads to Defective UDP-GlcNAc Biosynthesis A global combined network that connects metabolite nodes to reaction nodes was constructed based on the detected metabolites and the corresponding enzymes. The node of each reaction is connected to its respective substrates and products (solid circles). Enzymes that catalyze representative reactions are shown (diamonds). The relative levels of metabolites and enzymes in 20%OI ESCs compared to control ESCs is indicated by different colors (red, upregulated; green, downregulated; blue, not measured), and the significance is indicated by dot size (the bigger the more significant).

Figure S11. UDP-GlcNAc Links Oxidative Phosphorylation to Pluripotency

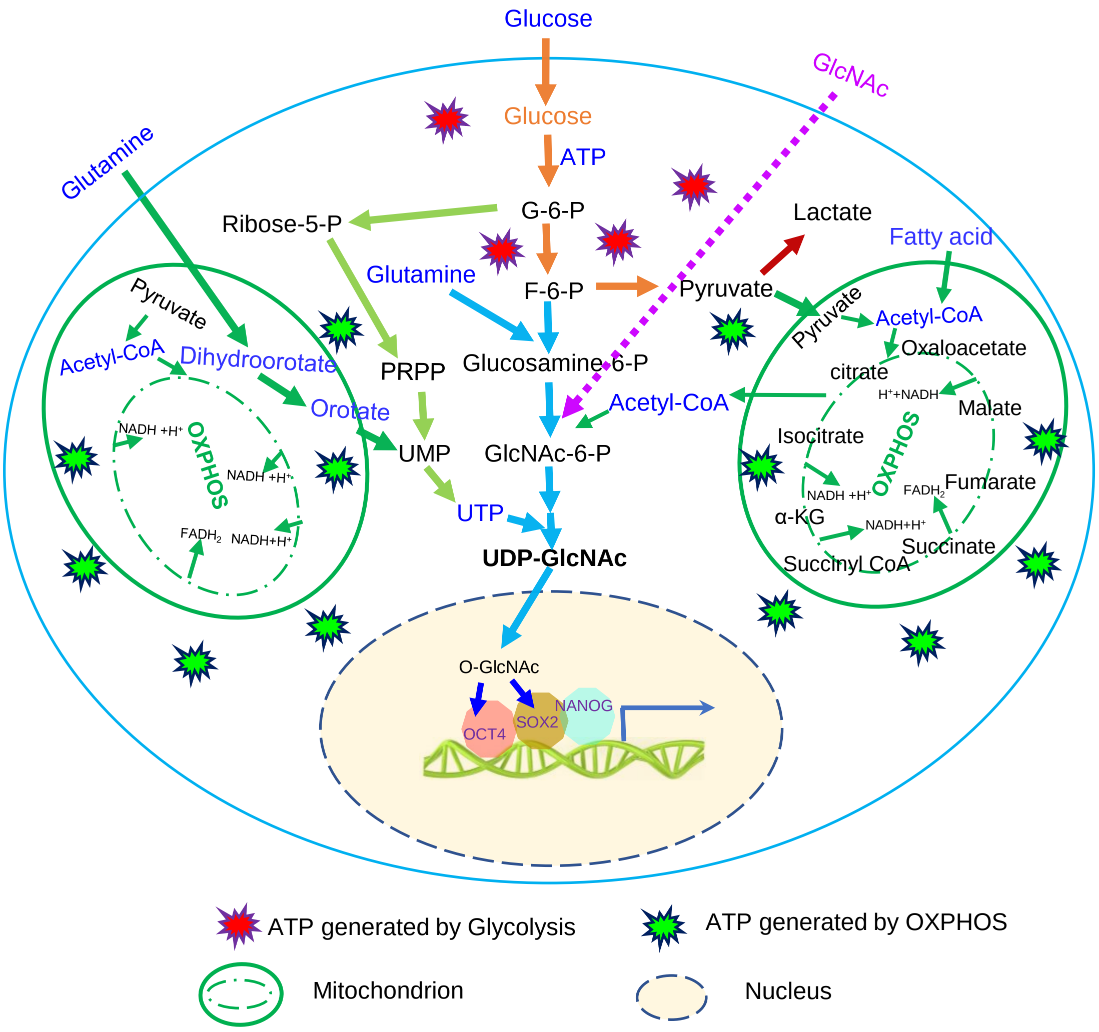

Figure S11. UDP-GlcNAc Links Oxidative Phosphorylation to Pluripotency ESC mitochondria have a high ATP generation capacity. Mitochondrial respiration produces the majority of cellular ATP in ESC, and couples with the hexosamine biosynthesis pathway to generate UDP-GlcNAc for O-GlcNAcylation of pluripotency factors like OCT4 and SOX2, thereby safeguarding ESC identity. Moderate inhibition of mitochondrial respiration leads to incomplete catabolism of glucose together with abnormal metabolism of glutamine, nucleotides, and acetyl-CoA, resulting in decreased UDP-GlcNAc generation and compromised pluripotency. This effect can be ameliorated by direct supplementation of GlcNAc.
